# Effects of rearing system and antibiotic treatment on immune function, gut microbiota and metabolites of broiler chickens

**DOI:** 10.1186/s40104-022-00788-y

**Published:** 2022-12-16

**Authors:** Bochen Song, Peng Li, Huiping Xu, Zhong Wang, Jianmin Yuan, Bingkun Zhang, Zengpeng Lv, Zhigang Song, Yuming Guo

**Affiliations:** 1 State Key Laboratory of Animal Nutrition, College of Animal Science and Technology, China Agricultural University, Beijing, 100193 China; 2 Department of Animal Science, Shandong Agricultural University, Taian, 271018 China

**Keywords:** Broad-spectrum antibiotics, Broiler chickens, Gut microbiota, Immune function, Metabolites, Rearing system

## Abstract

**Background:**

In China, cage systems with a high space utilization have gradually replaced ground litter systems, but the disease incidence of chickens in cages is higher. Broilers in the ground litter pens may be stimulated by more environmental microbes during the growth process and show strong immune function and status, but knowledge of which microbes and their metabolites play an immunomodulatory role is still limited. This study aimed to explore the differences and correlations in the immune function, gut microbiota and metabolites and the importance of gut microbiota of broilers raised in cages and ground litter pens.

**Methods:**

The experiment involved a 2 × 2 factorial arrangement, with rearing systems (cages or ground litter pens) and antibiotic treatment (with or without broad-spectrum antibiotics in drinking water) as factors.

**Results:**

The results showed that, compared with the cage group, the ground litter broilers had stronger nonspecific immune function (Macrophages% and NO in blood), humoral immune function (IgG in blood, LPS stimulation index in ileum) and cellular immune function (T%, Tc%, ConA stimulation index and cytokines in blood). Antibiotic (ABX) treatment significantly reduced nonspecific immune function (Macrophages% and NO in blood, *iNOS* and *Mucin2* mRNA expression in ileum), humoral immune function (IgG in blood and sIgA in ileum) and cellular immune function (T% and cytokines in blood, Th and Tc ratio, *TLRs* and cytokines mRNA expression in ileum). Furthermore, the ground litter broilers had higher α diversity of microbiota in ileum. The relative abundance of *Staphylococcus*, *Jeotgalicoccus*, *Jeotgalibaca* and *Pediococcus* in the ileum of ground litter broilers were higher. ABX treatment significantly reduced the α diversity of ileal microbiota, with less *Chloroplast* and *Mitochondria*. In addition, the levels of acetic acid, isobutyric acid, kynurenic acid and allolithocholic acid in the ileum of ground litter broilers were higher. Spearman correlation analysis showed that *Jeotgalibaca*, *Pediococcus*, acetic acid, kynurenic acid and allolithocholic acid were related to the immune function.

**Conclusions:**

There were more potential pathogens, litter breeding bacteria, short-chain fatty acids, kynurenine, allolithocholic acid and tryptophan metabolites in the ileum of broilers in ground litter pens, which may be the reason for its stronger immune function and status.

**Supplementary Information:**

The online version contains supplementary material available at 10.1186/s40104-022-00788-y.

## Introduction

Modern broilers are the products of breeding with the goal of rapid growth. In the process of selection, the immune function of broilers is a concession to growth performance, and the disease resistance of broilers is generally reduced [1]. Traditionally, broilers have been reared on the ground litter pens or free rage system. At present, in China, the multilayer cages system has gradually become a trend. The cage system separates chickens from manure, reducing the chance of chickens coming into contact with microbes from litter and manure. In the absence of appropriate microbial stimulation, the immune system development of chickens may be slower, and the immune function may be poor [2]. It was often discovered in production and scientific research that the incidences of leg abnormalities, foot abnormality, *Salmonella* colonization in the cecum and mortality of chickens raised in cages were significantly higher than those raised in ground litter pen [3–5]. Although its internal mechanism is not yet clear, and it is worthy of further study and discussion.

Most studies believe that the immune functions of animals grown under different rearing systems have certain differences. It has been reported that compared with cage laying hens, the SRBC antibody titer in the serum of laying hens in the ground litter group is higher [6]. Compared with caged chickens, ground litter broilers have higher levels of *IL-1β* and *IFN-γ* mRNA in the ileum [7]. Compared with indoor captive chickens, free-range and semi-stocked chickens have higher titers against Newcastle disease virus and infectious bronchitis virus in their peripheral blood [8]. However, some studies also have found that there is no significant difference in the number of peripheral blood leukocytes between cage and ground litter laying hens [9]. Compared with cage chickens, the numbers of eosinophils, lymphocytes, basophils and monocytes in the peripheral blood of broilers in the ground litter group were lower, as well as the phagocytic index and phagocytic activity [10]. In general, animals that grow in an outdoor environment with more microbes have stronger immune functions [11–13]. Without causing excessive inflammation such as allergies, more suitable microbes in the growing environment may stimulate the animal's immune system to develop faster, resulting in stronger immune function and disease resistance and lower disease susceptibility [14, 15].

There are many microbes living in the gut. These microbes are collectively called the gut microbiota, and most of the microbes are related to their hosts [16]. In recent years, the gut microbiota has received increasing attention and has been shown to affect the structure and function of the intestine, ferment indigestible food residues in the host’s intestines and drive the development of the immune system [17]. Appropriate microbial colonization is important for a well-functioning immune system. Studies have reported that microbial colonization is very important for the induction of T cell homeostasis in the intestine [18, 19]. In addition, the gut microbiota can affect the production of intestinal mucosal antibodies [20]. The interaction between the microbiota and the immune system explains to a certain extent the importance of exposure to microbes for immune development and immune response. It has been reported that the early existence of beneficial microbes in the intestine can promote resistance to pathogens by improving the health and integrity of the intestine and help improve the development of the immune system [21].

Studies have also shown that mice treated with ABX are gradually depleted of gut microbiota, with negative effects on the immune system. In the MLN and PP of mice, the proportion of Treg cells is reduced when the gut microbiota is depleted by treatment with broad-spectrum antibiotics [22]. With the number of gut microbes in the ABX-treated mice decreased significantly, the lack of gut microbial stimulation will lead to a decrease in the proportion of Tc (cytotoxic T) cells, Treg cells and DC cells in the intestine, colon, mesenteric lymph nodes and spleen, and the cytokines IFN-γ and IL produced by Th (helper T) cells-17, IL-22 and IL-10 also are reduced [23]. However, whether the effects of antibiotics on the immune system are mainly caused by changes in the microbiota and metabolites and related mechanisms remains unclear.

Growth environment may shape the composition of gut microbiota of animals. Even mice with the same parental generation may have different gut microbiota. This may be because their living environments and diet regimens are different, indicating that the environment and diet processing may be the main factors determining the gut microbiota [24]. Previous reports have shown that the housing conditions of mice and other animals can cause significant changes in the gut microbiota, which further confirms the importance of the living environment in shaping the gut microbiota [25–27].

Some studies have found that rearing systems have a great impact on the health and meat quality of poultry, including chickens and ducks [28, 29]. However, there is still a lack of research on the effects of rearing systems on the immune function and gut microbiota of broilers. There are only a few studies on the effects of rearing systems on gut microbiota of poultry. It has been found that gut microbiota of poultry that grows in an environment with more microbes have higher α diversity, many of which may come from grass and soil [30–32].

At the same time, most of the current comparative studies on rearing systems compare outdoor and indoor rearing systems. However, the temperature, humidity and light intensity in different indoor and outdoor rearing systems are different, and the factors affecting animal immune function and gut microbiota are more complicated. This also may be the reason for the inconsistent results of this type of research. Research in the same livestock house can eliminate most of these interference factors except environmental microbes. Therefore, this study explored the effect of adding broad-spectrum antibiotics to drinking water on the immune function and gut microbiota of broilers in the same chicken house with double-layer cages and ground litter rearing systems. It looked for the gut microbiota and its metabolites related to the immune function of broilers and determined if the internal mechanism of the immune function of broilers in the ground litter rearing system is stronger than that of caged broilers. This study provides a theoretical basis for regulating the immune function of poultry through nutritional means with gut microbiota as the target.

## Materials and methods

### Experimental animals and diets

A total of 444 Arbor Acres broilers (1-day-old) were randomly assigned to four groups using a 2 × 2 factor arrangement. There were 6 replicates in each treatment group, 10 chickens for each replicate cage, and 27 chickens for each repeat on the ground litter. The four groups were the cage control group (CC), the cage antibiotic group (CK), the ground litter control group (GC) and the ground litter antibiotic group (GK). The feed formula is shown in Table S1 (Additional file 1). According to the recommendations of the National Research Council (NRC 1994) [33], a drug-free corn-soybean meal diet is formulated to meet or exceed the nutritional requirements of broilers. Standard management procedures were used throughout the experiment. Chickens have free access to clean water and feed.

### Rearing systems

Standard management procedures were used throughout the experiment. The chicken cages were 100 cm in length × 70 cm in width × 40 cm in height, and the stocking density was 14.3 birds/m^2^. The ground chicken pens were 310 cm in length × 60 cm in width × 75 cm in height, and the stocking density was 14.4 birds/m^2^. According to the actual situation of the ground litter rearing system in the industry, we used rice husks as bedding litter, and stacked the rice husks to 8 cm in height until the end of the experiment. Double-layer cages and ground litter pens were located in the same chicken house and had the same levels of light, temperature and humidity. Samples were collected at d 10 and 28 to determine indicators, and the test period was 28 d.

### Establishment of a model of gut microbiota reduction

With reference to the experience of establishing a model to reduce the gut microbiota in mice [23], we use drinking water to add compound broad-spectrum antibiotics (neomycin, 1 g/L; ampicillin, 1 g/L; vancomycin, 0.5 g/L; metronidazole, 1 g/L). To establish a model of gut microbiota reduction, we calculate the water consumption by two times the feed intake, and we modulate the same concentration of compound broad-spectrum antibiotic drinking water into the bucket, according to daily water consumption. For the blank control group, we add the same volume of normal drinking water.

### Sample collection and index determination

#### Growth performance

The feed consumption and the bodyweight of the birds were measured in each cage and ground litter pen at d 10 and 28. The average daily gain (ADG), average daily feed intake (ADFI), and feed conversion ratio (FCR) were calculated from feed intake and body weight data. The feed wastage and bird mortality were recorded daily, and the feed consumption and FCR were adjusted for feed wastage and remaining birds.

#### Peripheral blood mononuclear cell isolation

The isolation of peripheral blood mononuclear cells (PBMC) was conducted as previously described using density gradient centrifugation with Ficoll-Paque Plus following the manufacturer’s guidelines [34]. Briefly, six healthy chickens (1 bird per replicate) were randomly selected from each treatment group on d 10 and 28. Heparinized blood samples were collected from the wing vein and then diluted 1:1 with sterile calcium- and magnesium-free Hank’s balanced salt solution (CMF-HBSS; H6648, Sigma-Aldrich, Saint Louis, MO, USA). The diluted samples were placed on ice and then carefully layered into a tube containing an equal volume of Ficoll lymphocyte separation medium (LDS1088C, Tianjin HaoYang Biological Manufacture Co., Ltd., Tianjin, China) to form a distinct layer above the Ficoll. Following centrifugation at 400 × *g* for 30 min at room temperature, the white flocculent material on the interface between the plasma and the lymphocyte separation medium was transferred to a clean tube using a sterile transfer pipette. The lymphocyte suspension was washed 3 times with RPMI 1640 (C11875500BT, Invitrogen Corp., Grand Island, NY, USA) incomplete culture medium and then resuspended in 2 mL of RPMI 1640 complete culture medium supplemented with 5% (vol/vol) fetal calf serum, 0.5% penicillin (final concentration, 100 U/mL), 0.5% streptomycin (final concentration, 100 μg/mL), and 1% N-(2-hydroxyethyl)-piperazine-N-2-ethanesulfonic acid (HEPES, final concentration, 24 mmol/L; Amresco 0511, Amresco Inc., Cleveland, OH, USA). The live cells were detected using the Trypan blue dye exclusion technique and a microscope (DM6000B, Leica Microsystems, Wetzla, Germany). The cell suspensions were diluted to a final concentration of 1 × 10^7^ cells/mL in RPMI 1640 medium for subsequent analysis.

#### Isolation of ileum propria lymphocytes (LPLs)

Separation solution was prepared by adding 5% FBS (10099141C, Gibco, Grand Island, NY, USA), 1 mmol/L DTT (BL552A, Biosharp, Hefei, Anhui, China) and 10 mmol/L HEPES (15630080, Gibco, Grand Island, NY, USA) to D-Hank's solution without calcium magnesium phenol red (H6648, Sigma-Aldrich, Saint Louis, MO, USA). Digestion solution was prepared by adding 5% FBS (10099141C, Gibco, Grand Island, NY, USA), 0.25% pancreatin (15090046, Gibco, Grand Island, NY, USA), 0.1% collagenase I (17100, Gibco, Grand Island, NY, USA) and 100 U/mL DNAse I (EN0521, Thermo Scientific, NY, USA) and incubating at 37 °C for 5 min.

For cleaning, 1 g of the anterior ileum (1 cm after the yolk antrum) was cut out. All samples were the same weight. The intestinal tube was cut longitudinally along the mesentery side. The small intestine was turned so that the mucosa faced outward, after which it was rinsed gently in Hank's until the chyme was completely washed away, and it was then cut into approximately 0.5 cm intestinal tissue fragments laterally. For separation, 5 mL of separation solution was added to a 50-mL centrifuge tube, the tube was placed in a constant temperature shaking box and shaken at 37 °C (250 r/min) for 15 min and vortexed for 30 s, and the intestinal tissue fragments were then filtered through a 200-mesh cell sieve, after which the intestinal tissue fragments were collected in a 50-mL centrifuge tube. To chop the intestinal tissue fragments, they were moved to a petri dish and ophthalmic scissors were used to cut the tissue 100 times into a muddy shape. 5 mL of digestion solution was then added to a 50-mL centrifuge tube, and the samples were placed in a constant temperature shaking box and shaken (250 r/min) at 37 °C for 30–45 min. The samples were then vortexed for 30 s, the intestinal tissue fragments were filtered through a 300-mesh (48 μm) cell sieve, and the filtrate was collected in a sterile 7-mL centrifuge tube and centrifuged at 4 °C (400 × *g* or 3000 r/min) 10 min. The supernatant was discarded, and the pellet was resuspended in 2 mL of RPMI 1640 (or D-Hank's, wash). Samples were then centrifuged (400 × *g* or 3000 r/min) at 4 °C for 10 min, the supernatant was discarded, and the pellet was resuspended in 2 mL of RPMI 1640. The organ tissue lymphocyte separation solution was extracted by differential centrifugation (2500 r/min), washed repeatedly, resuspended and finally cultured with complete RPMI 1640. According to the method of the organ lymphocyte extraction kit, Ficoll-Paque Plus (LDS1088C, Tianjin HaoYang Biological Manufacture Co., Ltd., Tianjin, China) was used for density gradient centrifugation, and the subsequent method was the same as that of peripheral blood lymphocyte separation.

#### Peripheral blood and ileum mononuclear cell proliferation

A 3-(4,5-dimethylthiazol)-2,5-diphenyltetrazolium bromide (MTT, Sigma Chemical Co., St. Louis, MO, USA) assay was used to determine the peripheral blood and ileum lymphocyte proliferation response. Briefly, 100 μL of the PBMCs suspension and 100 μL of RPMI 1640 in the absence or presence of 90 μg/mL concanavalin A (Con A; C2613, Sigma-Aldrich, Saint Louis, MO, USA) or 50 μg/mL lipopolysaccharide (L3129, Sigma-Aldrich, Saint Louis, MO, USA) were added to a 96-well microtiter plate (Costar 3599, Corning Inc., NY, USA). The cultures were set up in triplicate. After a 68-h incubation in a 5% CO_2_ incubator (MCO-18AIC CO_2_ incubator, Sanyo Electric Biomedical Co. Ltd., Tokyo, Japan) at 39 °C, MTT was added to each well at a final concentration of 5 mg/mL. The cells were incubated for an additional 4 h, and then, 100 μL of 10% sodium dodecyl sulfate dissolved in 0.04 mol/L HCl solution was added to each well to lyse the cells and solubilize the MTT crystals. Finally, the absorbance value of each sample was determined using an automated ELISA reader (model 550 Microplate Reader, Bio-Rad Pacific Ltd., Hongkong, China) at 570 nm. The stimulation index (SI) for each sample was calculated based on the following formula:

$$\mathrm{SI}\hspace{0.17em}=\hspace{0.17em}(\mathrm{Absorbance}\;\mathrm{value}\;\mathrm{of}\;\mathrm{mitogen}\;\mathrm{stimulated}\;\mathrm{cells})/(\mathrm{Absorbance}\;\mathrm{value}\;\mathrm{of}\;\mathrm{media}\;\mathrm{without}\;\mathrm{mitogen})$$

#### Phagocytic activity of mononuclear lymphocytes in peripheral blood and ileum

The phagocytic activity levels of peripheral blood and ileal mononuclear lymphocytes were measured by the neutral red assay method. In short, peripheral blood PBMCs were collected, and the number of cells was adjusted to 2 × 10^6^/mL with RPMI 1640 medium. Samples of one hundred microliters were then incubated in a 96-well cell culture plate for 2 h (with 3 replicate wells), the supernatant was discarded, 200 μL/well of 0.1% neutral red solution (N299163, Shanghai Aladdin Biochemical Technology Co., Ltd., Shanghai, China) was added, and the cells were further incubated for 2 h. The supernatant was then discarded, and any remaining neutral red was washed away with PBS (3 times). Cell lysate was added at 200 μL/well (ethanol:acetic acid = 1:1), kept in the dark at 4 ºC for 12 h, and the OD value was measured at 550 nm.

#### Determination of lymphocyte subsets in peripheral blood and ileum PBMCs by flow cytometry

As mentioned earlier [35, 36], flow cytometry was used to analyse the percentages of CD3^+^ , CD4^+^ , CD8^+^ and KUL01^+^ cells. In short, primary antibodies against mouse anti-chicken CD45-FITC (8270–02, Southern Biotech, Birmingham, AL, USA), mouse anti-chicken CD3-Alexa Fluor® 700 (8200–27, Southern Biotech, Birmingham, AL, USA), mouse anti-chicken CD4-APC (8210–11, Southern Biotech, Birmingham, AL, USA), mouse anti-chicken CD8α-Pacific Blue™ (8220–26, Southern Biotech, Birmingham, AL, USA) and mouse anti-chicken monocyte/macrophage-PE (8420–09, Southern Biotech, Birmingham, AL, USA) were used. A volume of 100 μL of PBMCs (2 × 10^6^ cells) was added to a 1.0-mL Eppendorf tube, and 25 μL of diluted primary monoclonal antibody (1:100 dilution) and negative isotype control IgG (mouse IgG1κ)-SPRD, mouse IgG1κ-FITC and mouse IgG1κ-R-PE) were used for staining. After incubating for 45 min at room temperature, the cells were washed twice with cold PBS and centrifuged at 1800 × *g* for 30 min to remove unbound primary antibody. A total of 300 μL of haemolysin solution diluted in PBS (1:25) was added to each tube. Finally, the cells were washed twice, and the final volume was 500 μL. Five-colour flow cytometry analysis was performed using a Coulter XL (Beckman Coulter, Inc., Pasadena, California, USA) at Xiyuan Hospital of Traditional Chinese Medicine, China Academy of Chinese Medical Sciences. Then, the percentages of CD3^+^ T, CD4^+^ T, CD8^+^ T and monocyte/macrophage cells in PBMCs and LPL were calculated.

#### Serum NO, lysozyme activity, cytokine, immunoglobulin and mucosal sIgA contents

A commercial ELISA kit (Genorise Scientific Inc., Glen Mills, PA, USA) was used to determine the serum levels of IL-1β, IL-4, IL-10, IL-17 and IFN-γ. According to the instructions, a chicken IgG ELISA kit (E30-104, Bethyl Laboratories Inc., Montgomery, TX, USA) was used to determine the level of IgG in the serum. Serum NO and lysozyme activities were measured using commercial ELISA kits (A013-2–1; A050-1–1, Nanjing Jiancheng Institute of Bioengineering, Nanjing, China). Ileal mucosa sIgA was measured using a commercial ELISA kit (YM-SQ2632, Shanghai Yuan Mu Biotechnology Co., Ltd., Shanghai, China).

#### Spleen and ileum gene expression

At d 10 and 28, molecular samples of the spleen and ileum were quick-frozen in liquid nitrogen and then transferred to a – 80 ºC low-temperature freezer to determine the expression levels of immune function-related genes in the spleen and ileum. TRIzol reagent was used to extract total ileum RNA, and a NanoDrop ultra-micro-calculation protein analyser was used to determine the quality and concentration of RNA. The reagent kit used in the reverse transcription step was the PrimeScript™ RT reagent Kit with gDNA Eraser (Perfect Real Time) from Takara. The cDNA obtained after reverse transcription was subjected to real-time fluorescent quantitative PCR on an ABI 7500 real-time fluorescent quantitative PCR instrument with the primers shown in Table S2 (Additional file 2). The fluorescence quantification kit was Takara's SYBR® Premix Ex Taq™ II (Tli RNaseH Plus), with GAPDH as the internal reference, and the results are expressed as 2 ^(−ΔΔCT)^.

#### Caecal microbial count

Under aseptic conditions, caecum specimens from broiler chickens on d 10 and 28 were collected, quickly frozen in liquid nitrogen and stored at – 20 °C for caecal bacterial count. The specific method was as follows: the caecum was placed on ice (approximately 4 °C) to thaw, and 0.3 g content was weighed on a balance on a clean bench and placed in a 5-mL sterile centrifuge tube. Then, 2.7 mL of sterile saline was added for a tenfold dilution, and the sample was shaken and mixed on a micro shaker and allowed to stand for 10 min. Then, 0.3 mL of the supernatant was moved to a sterile centrifuge tube, and sterile saline was used to perform gradient dilutions of 10^2^, 10^3^, 10^4^, 10^5^, 10^6^, 10^7^, 10^8^. 100 μL of the solution from each dilution tube was inoculated on the corresponding selective medium, and the plate was spread until the solution was dry on the medium. After culturing under the corresponding conditions, 30–300 colonies were selected for bacterial count. Among them, Aerobic bacteria was cultivated in nutrient agar medium (CM107, Beijing Luqiao Technology Co., Ltd., Beijing, China) and cultured under anaerobic conditions at 37 °C. Plates were counted after 24 h. Anaerobic bacteria were counted on anaerobic agar medium (CM1514, Beijing Luqiao Technology Co., Ltd., Beijing, China), and the culture conditions were 5% CO_2_ and 37 °C for 24 h. The logarithm of the number of bacteria per gram of caecal content (log_10_ CFU/g) was used to express the results.

#### DNA extraction and high-throughput sequencing

Bacterial DNA was extracted from ileal digesta with a QIAamp DNA Stool Mini Kit (Qiagen Inc., Valencia, CA, USA) according to the manufacturer’s protocol. The concentrations of DNA extracts were measured on a NanoDrop 2000 spectrophotometer (Thermo Scientific, MA, USA). The V3 and V4 region of the bacterial 16S rRNA gene was amplified with the barcoded primer pair 515F/806R (515F: 5′-GTG CCA GCM GCC GCG GTA A-3′, 806R: 5′-GGA CTA CHV GGG TWT CTA AT-3′) according to previously described methods [37]. After amplification, PCR products run on a 2% agarose gel and were purified using a QIAquick Gel Extraction Kit (Qiagen, Germany). Purified amplicons were pooled in equimolar amounts, and their paired-end reads were sequenced on an Illumina HiSeq2500 PE250 platform (Illumina, San Diego, USA) at Novogene Bioinformatics Technology Co. Ltd. (Beijing, China).

#### Sequence processing and bioinformatics analysis

Raw tags were generated by merging paired-end reads using FLASH software (v1.2.7) [38]. High-quality clean tags were obtained by QIIME (v1.7.0) analysis [39], and chimera sequences were removed to obtain effective tags by using the UCHIME algorithm [40]. Sequences were analyzed by UPARSE software (v7.0.1001) and clustered into operational taxonomic units (OTUs) at a similarity level of 97% [41]. Each OTU was annotated with the Greengenes database [42]. Rarefaction curve and Venn diagram were created using R software (v2.15.3). Analysis of microbial alpha diversity was conducted using QIIME software with Python scripts [39]. Beta diversity was evaluated by principal component analysis (PCA) to show the differences of bacterial community structures, and the significance of separation was tested via ANOSIM using R (v2.15.3). PICRUSt analysis was used to predict the functional potential of bacteria communities [43]. OTUs were normalized by copy number, and metagenome prediction was further categorized into Kyoto Encyclopedia of Genes and Genomes (KEGG) at levels 2 and 3 [44].

#### Ileal contents short-chain fatty acids

Ileal chyme (0.5 g) was weighed in a 10-mL plastic centrifuge tube, 8 mL of deionized water was added, and the samples were ultrasonically shaken for 30 min and centrifuged at 8000 r/min at 4 °C for 10 min. The supernatant was diluted ten times and filtered with a 0.22-μm filter. Twenty-five microlitres of the filtrate was collected, and an ICS-3000 high-performance ion chromatograph (Dionex, USA) was used to detect SCFAs and lactic acid by conductivity. Organic acids were separated under gradient conditions in an AS11 analytical column (250 mm × 4 mm) and a AG11 guard column: the gradient used potassium hydroxide as the carrier, 0–5 min, 0.8–1.5 mmol/L; 5–10 min, 1.5–2.5 mmol/L; 10–15 min, 2.5 mmol/L, flow rate 1.0 mL/min.

#### Ileal contents in the non-targeted metabolome

The sample detection and data analysis were all completed by Tianjin Nuohe Zhiyuan Technology Co., Ltd (Tianjin, China). The extraction process of metabolites in ileal contents was as follows: (1) 100 μL of ileal contents was taken, 350 μL of extraction solution (methanol:acetonitrile:water volume ratio = 2:2:1, both methanol and acetonitrile were chromatographic grade, purchased from Merck) was added, and then 20 μL of internal standard *L*-2-chlorophenylalanine (CAS#: 103,616–89–3, ≥ 98%; purchased from Shanghai Hengbai Biotechnology Co., Ltd.) was added vortexed and mixed for 30 s and sonicated for 10 min under ice water bath conditions. (2) The samples were incubated at – 20 ºC for 1 h and then centrifuged at 13,000 r/min for 15 min at 4 ºC. (3) After carefully removing 400 μL of supernatant to a 1.5-mL centrifuge tube, the extract was then dried in a vacuum concentrator. (4) Then, 100 μL of extract (equal volume of acetonitrile and water) was added to the dried extract for reconstitution, and the samples were vortexed for 30 s and ultrasonicated for 10 min in an ice water bath. (5) The samples were then centrifuged at 12,000 r/min at 4 ºC for 15 min. (6) Finally, 60 μL of supernatant was carefully removed and placed in a 2-mL injection bottle, and 10 μL of each sample was mixed into a quality control sample, while 60 μL was taken for testing.

The system was used to analyse the metabolome components of the ileum content using an Agilent 1290 ultrahigh-performance liquid chromatograph in a series ABSciexTripleTOF6600 high-resolution mass spectrometer. The column used was an Acquity UPLC BEH Amide column (2.1 mm × 100 mm, 1.7 μm particle size) purchased from Waters (Milford, MA, USA). The mobile phase conditions were 25 mmol/L ammonium acetate and 25 mmol/L ammonia solution (A) and 100% acetonitrile (B). The gradient items for the analysis of the ileal contents sample were: 0–0.5 min, 5% A, 95% B; 0.5–7 min, 5%–35% A, 95%–65% B; 7–8 min, 35%–60% A, 65%–40% B; 8–9 min, 60% A, 40% B; 9–9.1 min, 60%–5% A, 40%–95% B; 9.1–12 min, 5% A, 95% B; the flow rate was 0.5 mL/min, and the injection volume was 2 μL. The mass spectrometry conditions were as follows: AB6600TripleTOF mass spectrometer, which can perform primary and secondary mass spectrometry data based on the IDA function under the control of the software (AnalystTF1.7, ABSciex). In each data acquisition cycle, the strongest signals with greater than 100 molecules are screened out. The ions were collected corresponding to the secondary mass spectrum data. Bombardment energy: 35 eV, 15 secondary spectra every 50 ms. The ESI ion source parameters were set as follows: atomization pressure (GS1): 60 Pa, auxiliary pressure: 60 Pa, air curtain pressure: 30 Pa, temperature: 550ºC, and spray voltage: 5500 V (positive ion mode) or –4500 V (negative ion mode). QC samples with RSD < 30% were screened, and a feature yield rate > 80% was required to ensure good system stability.

The data were first converted to mzXML format using MSconventer, and XCMS (XCMS1.41.0) was used for peak search, peak alignment and other data processing. Then, the data processing and matching of substance identification were performed, and the xcms4dda and xcms4lipid developed by Nuovo Zhiyuan based on XCMS were used. The program and self-built library were processed, minfrac was set to 0.5, and the cut-off was set to 0.8. First, the secondary data were screened. The screening principle is that as long as one forward and one reverse signal is identified, the peak is retained. Second, the peaks of the first- and second-level data are matched, that is, the peaks of the first-level data corresponding to the second-level data are found, and matching is performed according to mztolerance ± 25 ppm. Data analysis included three parts: basic data analysis and personalized data analysis. The goal of basic data analysis is to perform univariate analysis (UVA) and multivariate analysis (MVA) on the qualitative and quantitative results of the metabolome to screen for metabolites with significant differences. Univariate statistical analysis includes data preprocessing (first simulating the missing values in the original data; the numerical simulation method is the minimum one-half method to fill, and then using the total ion current of each sample for normalization), Student’s *t*-test and analysis of variance. Multivariate statistical analysis includes principal component analysis (PCA) and partial least squares regression analysis (Partial Least Squares Discrimination Analysis, PLS-DA), with differential compound screening and identification. Personalized data analysis is a series of bioinformatics analyses on metabolites with significant differences on the basis of basic data analysis, including KEGG annotation analysis of different metabolites and metabolic pathway analysis.

### Statistical analysis

SPSS 20.0 software was used to perform the statistical analysis on each group of data. The GLM process was used for statistical analysis. When the interaction was significant, one-way analysis of variance was used, and Duncan’s multiple comparison analysis was used for differences between treatments. *P* < 0.05 was considered significant, and *P* values between 0.05 and 0.10 were classified as trends. The spearman rank correlation coefficient was used for the evaluation of the correlation analysis of the variables and microbes in the broiler chickens.

## Results

### Growth performance and immune organ index

**Fig. 1:**
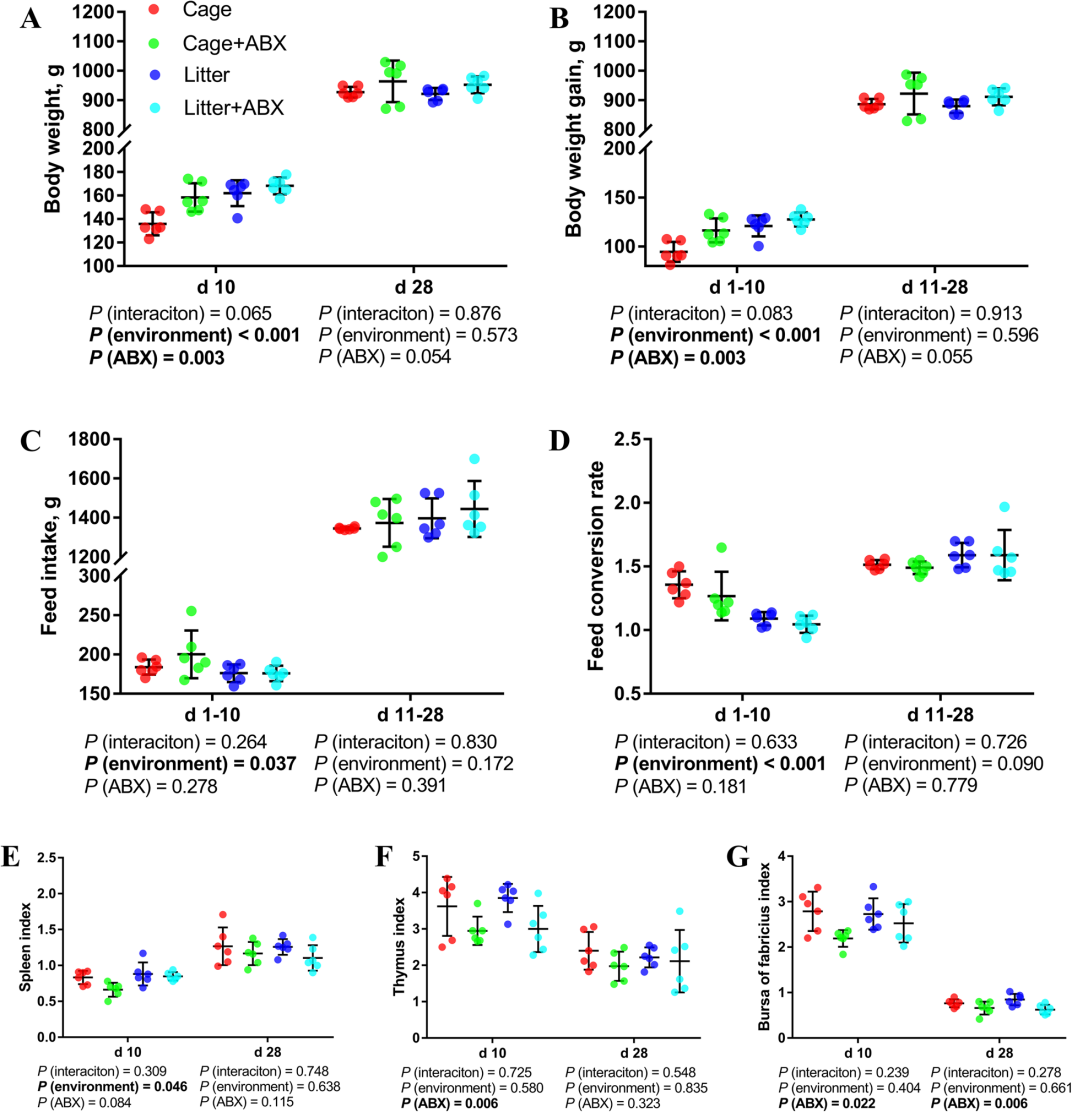
Effects of ABX treatment on the growth performance, mortality rate and immune organ index of broiler chickens with cages and ground litter floor pens. The body weight (**A**), body weight gain (**B**), feed intake (**C**) and feed conversion ratio (**D**) were analyzed by weighted. The spleen index (**E**), thymus index (**F**) and bursa of fabricius index (**G**) were analyzed by weighted. All graphs are presented as mean, with the standard deviation (SD) shown via the whiskers. The main effect and interaction effects were analyzed using the general linear model (GLM) procedure, with the *P* values for the main effects written out below each plot. The one-way ANOVA and multiple comparisons were performed when interactive effects differed significantly. Cage: cage control group; Cage + ABX: cage with ABX group; Litter: ground litter control group; Litter + ABX: ground litter with ABX group

As shown in Fig. 1 and Table S3 (Additional file 3), compared with the cage group, ground litter broilers had higher average body weight at d 10, higher feed intake and body weight gain at d 0–10 (*P* < 0.05), and lower FCR at d 0–10 (*P* < 0.05). However, the difference was not significant at d 28. Compared with the control group, ABX treatment significantly increased the average body weight of broilers on d 10 and body weight gain on d 0–10 (*P* < 0.05). Compared with the cage group, ground litter broilers had a higher d 10 spleen index (*P* < 0.05). Compared with the control group, ABX treatment significantly reduced the d 10 thymus index and the d 10 and d 28 bursal indices (*P* < 0.05), and it had a tendency to decrease the spleen index at d 10 (0.05 < *P* < 0.1).

### Peripheral nonspecific immune function and humoral immune function

**Fig. 2:**
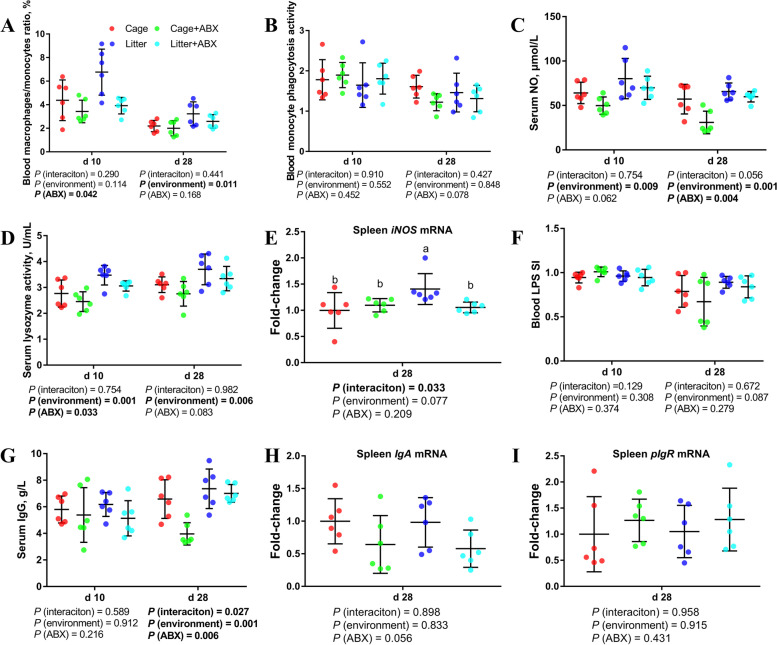
Effects of ABX treatment on peripheral nonspecific immune function and humoral immune function of broiler chickens with cages and ground litter floor pens. The frequencies of Mononuclear/Macrophage (**A**) of peripheral blood lymphocytes were analyzed by flow cytometry. The phagocytic activity of Monocytes (**B**) was analyzed by neutral red method. The levels of NO (**C**), lysozyme activity (**D**), and IgG (**G**) were analyzed by ELISA kit. Peripheral blood lymphocytes were stimulated with lipopolysaccharide (LPS) (**F**), and the stimulation index (SI) was calculated as described in the Materials and methods section. The mRNA levels of *iNOS* (**E**), IgA (**H**) and *pIgR* (**I**) in spleen were analyzed by RT-PCR. All graphs are presented as mean, with the standard deviation (SD) shown via the whiskers. The main effect and interaction effects were analyzed using the general linear model (GLM) procedure, with the *P* values for the main effects written out below each plot. The one-way ANOVA and multiple comparisons were performed when interactive effects differed significantly. The lowercase letters on the bar charts indicate significant differences (*P* < 0.05). Cage: cage control group; Cage + ABX: cage with ABX group; Litter: ground litter control group; Litter + ABX: ground litter with ABX group

As shown in Fig. 2, compared with cage broilers, the proportion of Mononuclear/Macrophages was higher on d 28 (*P* < 0.05). Compared with the control group, the proportion of peripheral blood Mononuclear/Macrophages in broilers on d 10 was lower after ABX treatment (*P* < 0.05). Compared with the control group, the phagocytic function of peripheral blood mononuclear cells in broilers on d 28 tended to decrease after ABX treatment (0.05 < *P* < 0.1). Compared with the cage group, the serum NO and lysozyme levels of d 10 and d 28 ground litter broilers were higher (*P* < 0.05). Compared with the normal group, the serum lysozyme levels of broilers on d 10 and d 28 were lower after ABX treatment (*P* < 0.05), and the d 28 NO level was lower (*P* < 0.05).

Compared with cage broilers, the peripheral blood B cell proliferation activity of ground litter broilers tended to increase on d 28 (0.05 < *P* < 0.1). Compared with the cage group, the serum IgG levels in ground litter broilers on d 28 was higher (*P* < 0.05). Compared with the normal group, the serum IgG levels in broiler chickens on d 28 was lower after ABX treatment (*P* < 0.05). The rearing system and antibiotic treatment had an interactive effect on the serum IgG level of broilers on d 28 (*P* < 0.05). Antibiotic treatment reduced IgG in cage broilers but had no significant effect on ground litter broilers.

### Peripheral cellular immune function

**Fig. 3:**
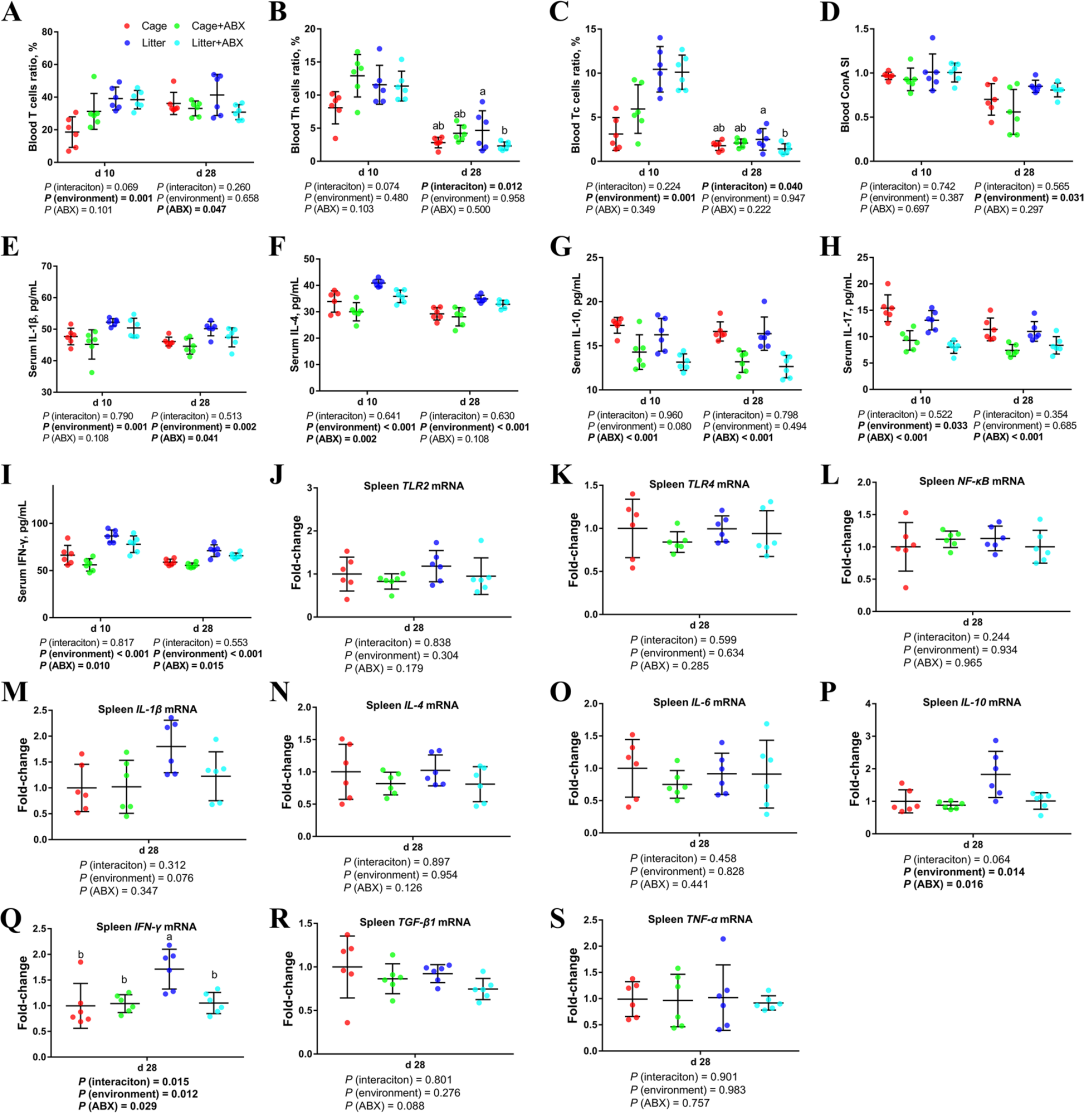
Effects of ABX treatment on peripheral cellular immune function of broiler chickens with cages and ground litter floor pens. The frequencies of T (**A**), Th (**B**) and Tc (**C**) of peripheral blood lymphocytes were analyzed by flow cytometry. Peripheral blood lymphocytes were stimulated with concanavalin A (ConA) (**D**), and the stimulation index (SI) was calculated as described in the Materials and methods section. The levels of IL-1β (**E**), IL-4 (**F**), IL-10 (**G**), IL-17 (**H**) and IFN-γ (**I**) in serum were analyzed by ELISA kit. The mRNA levels of *TLR2* (**J**), *TLR4* (**K**), *NF-κB* (**L**), *IL-1β* (**M**), *IL-4* (**N**), *IL-6* (**O**), *IL-10* (**P**), *IFN-γ* (**Q**), *TGF-β1*(**R**) and *TNF-α* (**S**) in spleen were analyzed by RT-PCR. All graphs are presented as mean, with the standard deviation (SD) shown via the whiskers. The main effect and interaction effects were analyzed using the general linear model (GLM) procedure, with the *P* values for the main effects written out below each plot. The one-way ANOVA and multiple comparisons were performed when interactive effects differed significantly. The lowercase letters on the bar charts indicate significant differences (*P* < 0.05). Cage: cage control group; Cage + ABX: cage with ABX group; Litter: ground litter control group; Litter + ABX: ground litter with ABX group

As shown in Fig. 3, compared with cage broilers, the proportion of peripheral blood T cells and Tc cells in d 10 ground litter broilers was higher (*P* < 0.05). Compared with the control group, the proportion of peripheral blood T cells in broilers on d 28 was lower after ABX treatment (*P* < 0.05). Compared with cage broilers, d 28 ground litter broilers had higher peripheral blood T cell proliferation activity (*P* < 0.05).

Compared with the cage group, the serum IL-1β, IL-4 and IFN-γ levels of broilers in the ground litter groups on d 10 and d 28 were higher (*P* < 0.05), and the serum IL-17 level on d 10 was higher (*P* < 0.05). Compared with the normal group, the serum IL-4, IL-10, IL-17 and IFN-γ levels of broilers on d 10 and d 28 were lower after ABX treatment (*P* < 0.05), and the serum IL-1β, IL-10, IL-17 and IFN-γ levels in broiler chickens on d 28 were lower (*P* < 0.05).

Compared with cage broilers, d 28 ground litter broilers had higher levels of *IFN-γ* and *IL-10* mRNA in the spleen (*P* < 0.05), and *IL-1β* tended to increase (0.05 < *P* < 0.1). Compared with the control group, the mRNA levels of *IFN-γ* and *IL-10* in the spleen of broilers on d 28 were lower after ABX treatment (*P* < 0.05), and *TGF-β1* and *IgA* had a decreasing trend (0.05 < *P* < 0.1).

### Intestinal nonspecific immune function and humoral immune function

**Fig. 4:**
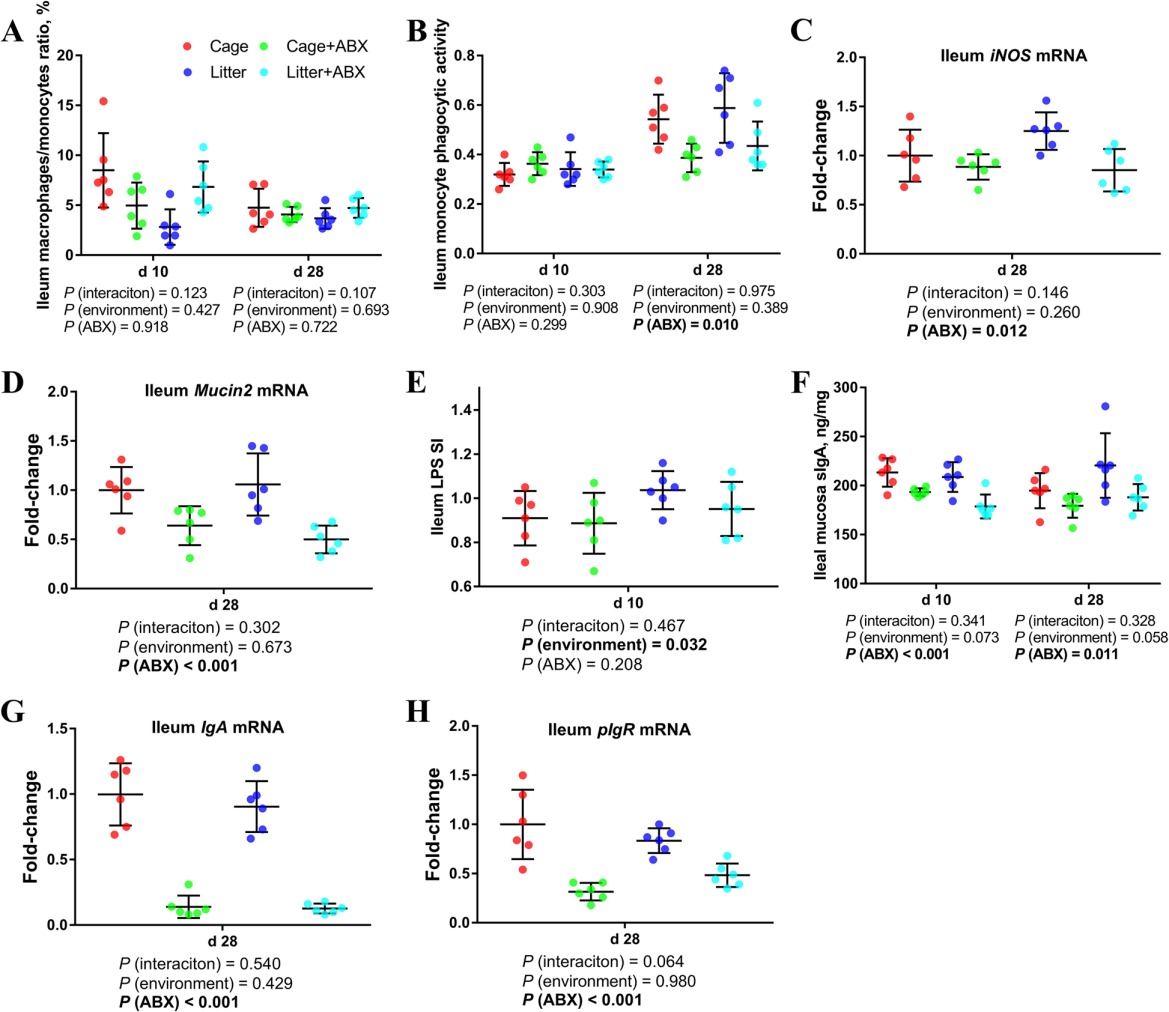
Effects of ABX treatment on intestinal nonspecific immune function and humoral immune function of broiler chickens with cages and ground litter floor pens. The frequencies of mononuclear/macrophage (**A**) of ileum lymphocytes were analyzed by flow cytometry. The phagocytic activity of monocytes (**B**) was analyzed by neutral red method. The mRNA levels of *iNOS* (**C**), *Mucin-2* (**D**), *IgA* (**G**) and *pIgR* (**H**) in jejunum and ileum were analyzed by RT-PCR. Ileum lymphocytes were stimulated with lipopolysaccharide (LPS) (**E**), and the stimulation index (SI) was calculated as described in the Materials and methods section. The level of sIgA of ileum mucosa (**F**) was analyzed by ELISA kit. All graphs are presented as mean, with the standard deviation (SD) shown via the whiskers. The main effect and interaction effects were analyzed using the general linear model (GLM) procedure, with the *P* values for the main effects written out below each plot. The one-way ANOVA and multiple comparisons were performed when interactive effects differed significantly. Cage: cage control group; Cage + ABX: cage with ABX group; Litter: ground litter control group; Litter + ABX: ground litter with ABX group

As shown in Fig. 4, compared with the control group, the phagocytic ability of ileal monocytes in broilers on d 28 was lower after ABX treatment (*P* < 0.05). Compared with the normal group, the ABX treatment significantly reduced ileal *iNOS* and *Mucin2* mRNA levels (*P* < 0.05).

Compared with cage broilers, the ileal B cell proliferation activity of d 10 ground litter broilers was higher (*P* < 0.05). There was no significant difference in sIgA levels in the ileal mucosa of cage and ground litter broilers. Compared with the control group, the level of sIgA in the ileal mucosa of broilers was significantly decreased after ABX treatment (*P* < 0.05). Compared with the normal group, the ABX treatment significantly reduced ileal *IgA* and *pIgR* mRNA levels (*P* < 0.05).

### Intestinal cellular immune function

**Fig. 5:**
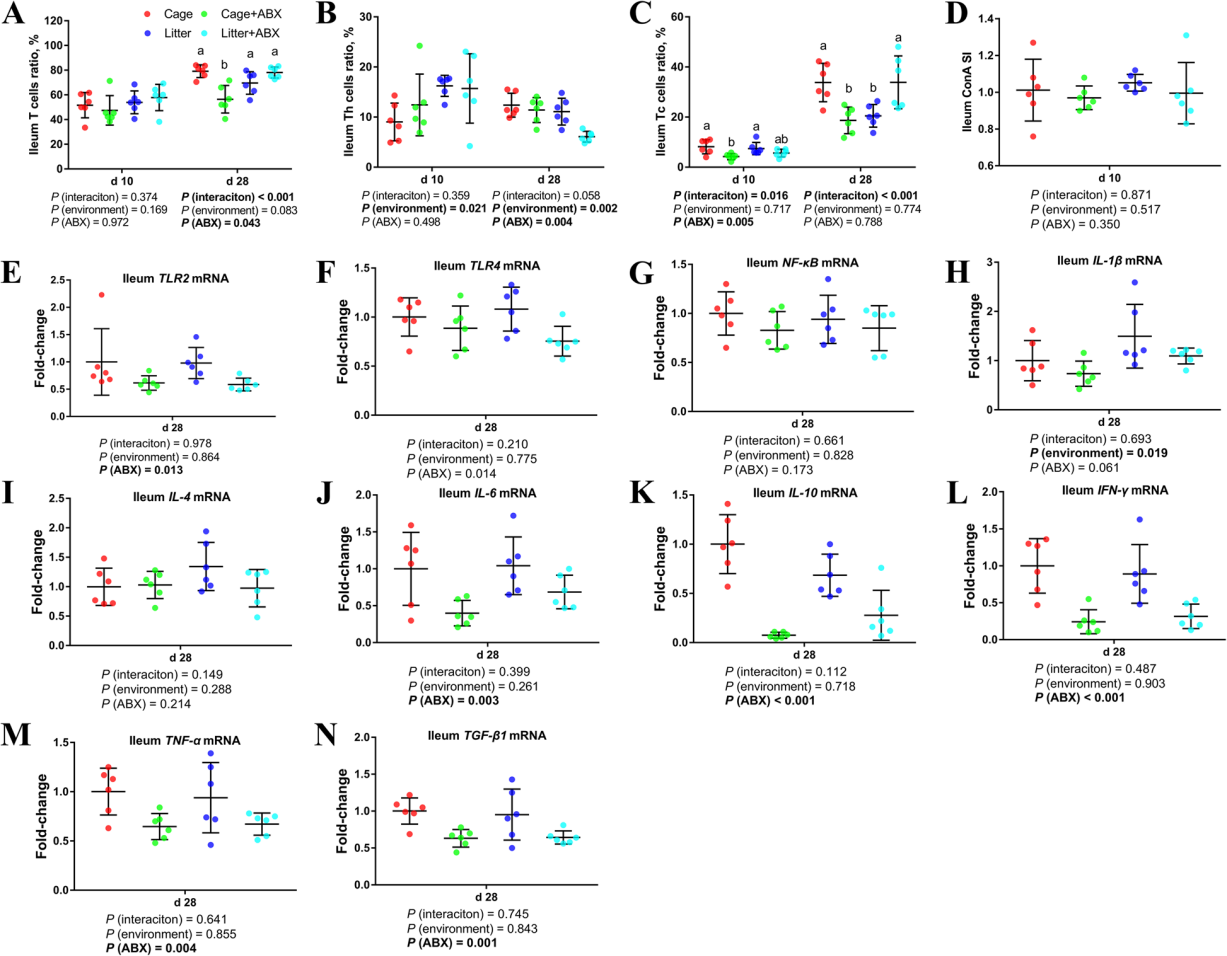
Effects of ABX treatment on intestinal cellular immune function of broiler chickens with cages and ground litter floor pens. The frequencies of T (**A**), Th (**B**) and Tc (**C**) of ileum lymphocytes were analyzed by flow cytometry. Ileum lymphocytes were stimulated with concanavalin A (ConA) (**D**), and the stimulation index (SI) was calculated as described in the Materials and methods section. The mRNA levels of *TLR2* (**E**), *TLR4* (**F**), *NF-κB* (**G**), *IL-1β* (**H**), *IL-4* (**l**), *IL-6* (**J**), *IL-10* (**K**), *IFN-γ* (**L**), *TNF-α* (**M**) and *TGF-β1* (**N**), in ileum were analyzed by RT-PCR. All graphs are presented as mean, with the standard deviation (SD) shown via the whiskers. The main effect and interaction effects were analyzed using the general linear model (GLM) procedure, with the *P* values for the main effects written out below each plot. The one-way ANOVA and multiple comparisons were performed when interactive effects differed significantly. The lowercase letters on the bar charts indicate significant differences (*P* < 0.05). Cage: cage control group; Cage + ABX: cage with ABX group; Litter: ground litter control group; Litter + ABX: ground litter with ABX group

As shown in Fig. 5, compared with cage broilers, d 10 ground litter broilers had a higher percentage of Th cells in the ileum (*P* < 0.05), a lower percentage of d 28 Th cells (*P* < 0.05), and an increased percentage of d 28 T cells (0.05 < *P* < 0.1). Compared with the control group, after ABX treatment, the proportion of Tc cells in the ileum of broilers on d 10 was lower (*P* < 0.05), and the proportion of T cells and Th cells on d 28 was lower (*P* < 0.05).

Compared with the cage group, broilers in the ground litter group had significantly increased ileal *IL-1β* mRNA levels on d 28 (*P* < 0.05). Compared with the normal group, the ABX treatment significantly reduced *TLR2, TLR4, IL-6, IL-10, IFN-γ, TNF-α* and *TGF-β1* mRNA levels in the ileum(*P* < 0.05).

### Ileum microbiota

#### Beta diversity

**Fig. 6:**
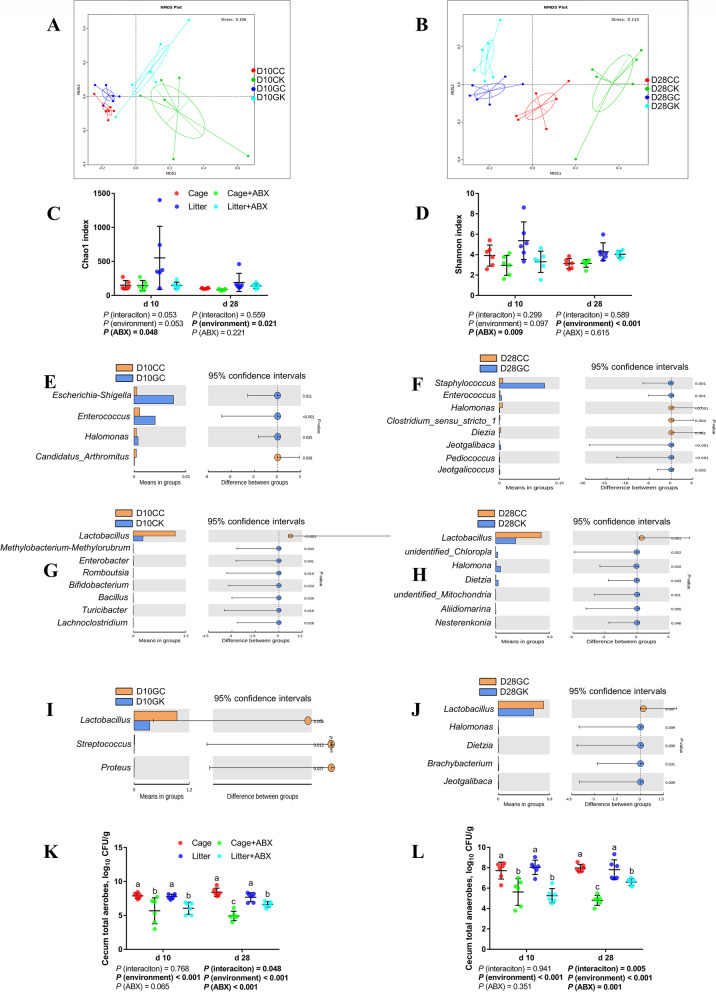
Effects of ABX treatment on the gut microbiota of broiler chickens with cages and ground litter floor pens. The beta diversity (**A**, **B**) of ileum microbiota of broiler chickens was analyzed by non-metric multidimensional scaling (NMDS). The alpha diversity of ileum microbiota of broiler chickens was analyzed by Chao1 index (**C**) and Shannon index (**D**). Differential microbes in ileum of broiler chickens by *T*-test (**E-J**). The numbers of aerobes (**K**) and anaerobes (**L**) in cecum were detected by culture count method. All graphs are presented as mean, with the standard deviation (SD) shown via the whiskers. The main effect and interaction effects were analyzed using the general linear model (GLM) procedure, with the *P* values for the main effects written out below each plot. The one-way ANOVA and multiple comparisons were performed when interactive effects differed significantly. The lowercase letters on the bar charts indicate significant differences (*P* < 0.05). CC/Cage: cage control group; CK/Cage + ABX: cage with ABX group; GC/Litter: ground litter control group; GK/Litter + ABX: ground litter with ABX group

Figure 6A and B shows that the four groups of CC, CK, GC and GK on d 10 and d 28 can be separated well, which proves that the ileal microbial composition of cage and ground litter broilers on d 10 and d 28 is significantly different. The ABX treatment can have a significant impact on the microbial composition of the ileum and increase the variation in the microbial composition.

#### Alpha diversity

As shown in Fig. 6C and D, compared with cage broilers, ground litter broilers had higher ileal microbial α diversity (*P* < 0.05), d 28 Shannon, Simpson and Chao1 indices were higher (*P* < 0.05), and d 10 observed species, Shannon and Chao1 had an increasing trend (0.05 < *P* < 0.1). Compared with the control group, the ileal microbial α diversity of broilers after ABX treatment decreased, the d 10 observed_species, Shannon and Chao1 indices were significantly decreased (*P* < 0.05), and the Simpson index had a decreasing trend (0.05 < *P* < 0.1).

#### Top 10 microbes in the ileum

Fig. S1 (Additional file 7) shows that compared with cage broilers, ground litter broilers significantly increased the relative abundance of d 10 *Escherichia-Shigella*, d 10 *Enterococcus*, and d 28 *Staphylococcus* in the ileum (*P* < 0.05), and significantly reduced the relative abundance of d 28 *Escherichia-Shigella*. The ABX treatment significantly increased the relative abundance of *Escherichia-Shigella*, *Chloroplast*, *Halomonas*, *Clostridium_sensu_stricto_1* and *Dietzia* in the ileum on d 10 and d 28 (*P* < 0.05), and significantly reduced the relative abundance of *Lactobacillus* on d 10 and d 28 (*P* < 0.05).

#### *T*-test for different microbes

It can be seen from Fig. 6E and F that compared with the cage group, ground litter broiler ileum significantly increased d 10 *Escherichia-Shigella*, d 10 *Enterococcus*, d 10 *Halomonas*, d 28 *Staphylococcus*, d 28 *Enterococcus*, d 28 *Jeotgalibaca*, d 28 *Pediococcus* and d 28 *Jeotgalicoccus* (*P* < 0.05), and significantly reduced d 10 *Candidatus_Arthromitus*, d 28 *Halomonas*, d 28 *Clostridium_sensu_stricto_1 * and d 28 *Dietzia* (*P* < 0.05).

As shown in Fig. 6G and H, compared with the cage blank group, ABX treatment significantly reduced on d 10 and d 28 *Lactobacillus* (*P* < 0.05), and significantly increased d 10 *Methylobacterium-Methylorubrum*, d 10 *Enterobacter*, d 10 *Romboutsia*, d 10 *Bifidobacterium*, d 10 *Bacillus*, d 10 *Turicibacter*, d 10 *Lachnoclostridium*, d 10 *unidentified_Chloroplast*, d 28 *Mitochondria*, d 28 *Halomonas*, d 28 *Dietzia*, d 28 *Aliidiomarina* and d 28 *Nesterenkonia* (*P* < 0.05).

Figure 6I and J show that, compared with the ground litter control group, the ABX treatment significantly reduced d 10 and d 28 *Lactobaclillus* (*P* < 0.05), and significantly increased d 10 *Streptococcus*, d 10 *Proteus*, d 28 *Halomonas*, d 28 *Dietzia*, d 28 *Brachybacterium* and d 28 *Jeotgalibaca* (*P* < 0.05).

### Caecal microbiota

As shown in Fig. 6K and L, compared with the cage group, the total aerobes and total anaerobes in the cecum of broilers in the d 28 ground litter group were higher (*P* < 0.05). Compared with the normal group, the numbers of total aerobes and total anaerobes in the cecum of broilers in the d 10 and d 28 broilers were significantly reduced after ABX treatment (*P* < 0.05).

### Nontargeted metabolome analysis of ileum contents

#### Data quality assessment and comparison of differences between groups

**Fig. 7:**
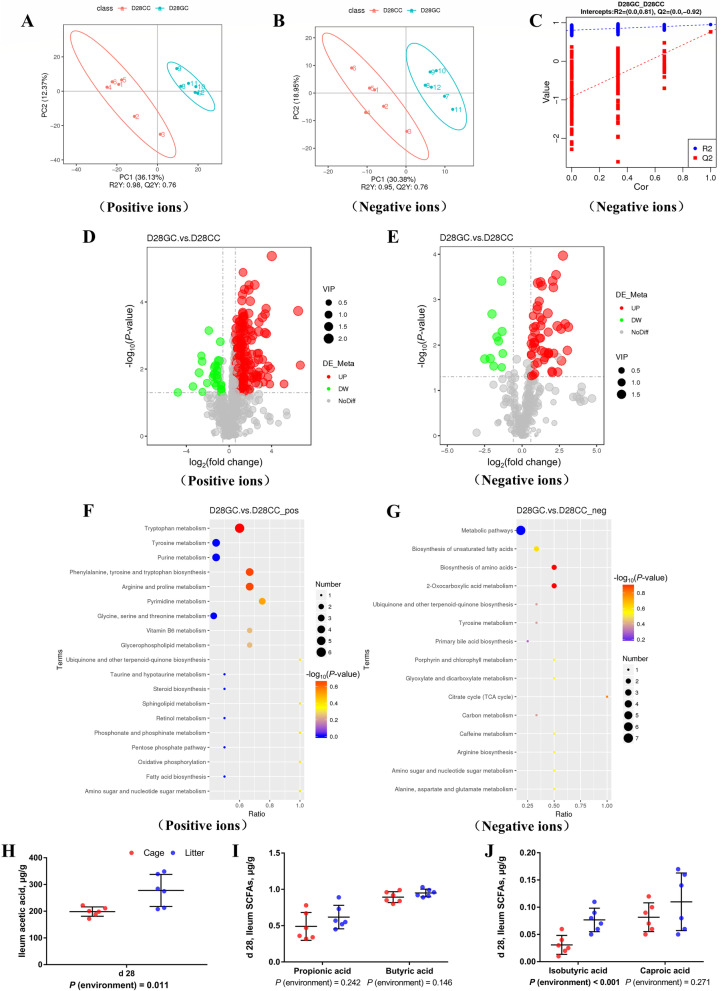
Differences in the ileal metabolome and short-chain fatty acids of broiler chickens with cages and ground litter floor pens. PLS-DA scatter plot for GC group vs. CC group on d 31 (**A, B**). Permutation test (**C**) of PLS-DA model for group GC versus CC (negative ion mode). Volcano plot for group GC versus CC (**D**, **E**). Each point in the mountain chart represents a metabolite, the abscissa represents the fold change (take the log2 logarithm) of each substance in the group, and the ordinate represents the *P*-value of the *t*-test (take the log10 logarithm). The size of the scatter points represents the VIP value of the PLS-DA model, and the larger the scatter point, the greater the VIP value. Scattered colors represent the final screening results. Significantly up-regulated metabolites are shown in red, significantly down-regulated metabolites are shown in green, and non-significantly different metabolites are shown in grey. GC group versus CC group KEGG pathway enrichment analysis bubble chart (**F**, **G**). According to the results of KEGG enrichment, select the top 20 pathways sorted by *P*-values from small to large to draw a bubble chart. The abscissa is *x*/*y* (the number of differential metabolites in the corresponding metabolic pathway/the total number of metabolites identified in the pathway), the larger the value, the higher the enrichment of differential metabolites in the pathway, and the ordinate is the KEGG pathway name. The ordinate and color of the bubble indicate the *P*-values of the enrichment analysis (take the negative common logarithm, ie –log_10_ *P*-values). The redder the color, the smaller the *P*-values, indicating that the degree of enrichment is more significant, the reliability of the test is greater and the difference in statistics is more significant. The size of the dot represents the number of different metabolites in the corresponding pathway. The larger the dot, the more differential metabolites in the pathway. The levels of short-chain fatty acids (**H-J**) in the ileal contents of broiler chickens were analyzed by high performance liquid chromatography (Dionex, USA). All graphs are presented as mean, with the standard deviation (SD) shown via the whiskers. Statistical differences were determined by one-way ANOVA, with the *P* values for the main effects written out below each plot. *P*-values represent the effect of the rearing system. GC/Litter: ground litter control group, CC/Cage: cage control group

To ensure the reliability of the data, the influence of factors such as the sample preparation process and instrument instability must be minimized. In the process of detection and analysis, three QC samples were used to monitor and ensure the stability of the instrument. We simulated the missing values in the original data and standardized the data. After obtaining the sorted data, we performed principal component analysis (PCA) and partial least squares discrimination analysis (PLS-DA) to obtain the blank GC group pair cage for the ground litter. The PLS-DA of the blank CC group has a scatter plot. Figure 7A, 7B and Fig. S2 (Additional file 8) show that compared with the cage blank group (CC), the ground litter blank group (GC) broiler ileum metabolite composition changed significantly, and there were significant differences between the two treatment groups. The permutation test result of the PLS-DA model is shown in Fig. 7C, the intercept R2 = 0.81, and Q2 = – 0.92. It can be seen that the PLS-DA model does not have an overfitting phenomenon, and the model has good robustness.

#### Differential metabolite screening between groups

According to the relative molecular mass and the secondary mass spectrometry data combined with the HMDB, PubChem and KEGG databases, a comparison search was carried out to analyze the potential differences in metabolites. According to the conditions that the variable importance in projection (VIP) of the first principal component of the PLS-DA model was greater than 1 and *P* < 0.05, 240 different metabolites were identified in positive ion mode (Table S4, Additional file 4), and 62 kinds were identified in negative ion mode (Table S5, Additional file 5). Compared with the cage group, the metabolites in the ileum content of ground litter broilers increased in concentration, mainly including kynurenic acid, *L*-kynurenine, allolithocholic acid, tauroursodeoxycholic acid sodium salt, creatinine, neopterin, aflatoxin G2, anthranilic acid, xanthurenic acid, octopine, agmatine, pyridoxine, citric acid, oleanolic acid, asiaticoside, ferulic acid, lactitol, glutaconic acid, perillartine, *D*-erythrose 4-phosphate and 264 other metabolites, while there were reductions in choice, indole, jerchol, 7-ketodeoxyvineic acid and 38 metabolites, including taurodeoxycholic acid, dehydrocholic acid, sodium dehydrocholate, tauroursodeoxycholic acid dihydrate, tauroursodeoxycholic acid sodium salt, deoxycholic acid, anthranilic acid, *L*-phenylalanine, glycocholic acid and thymine. The screening of differential metabolites was visualized in the form of a volcano map (Fig. 7D and E).

#### Differential metabolite KEGG analysis

Table S6 (Additional file 6) shows that the differential metabolites between groups were mapped to the biosynthesis of unsaturated fatty acids, tryptophan metabolism, arginine biosynthesis, taurine and hypotaurine metabolism, primary bile acid biosynthesis, amino sugar and nucleotide sugar metabolism and retinol in the KEGG database. There were 34 metabolic pathways, including metabolism; vitamin B_6_ metabolism; alanine, aspartate and glutamate metabolism; glycine, serine and threonine metabolism; tyrosine metabolism; oxidative phosphorylation; pentose phosphate pathway; citrate cycle (TCA cycle); and caffeine metabolism.

#### Enrichment analysis of metabolic pathways of differential metabolites

The metabolites of the different groups were entered into MetaboAnalyst, the database corresponding to the chicken used to perform enrichment analysis on the metabolic pathways, and the corresponding *P* value was calculated. Through topological analysis and enrichment analysis of the metabolic pathways where the differential metabolites were located, the metabolic pathways were screened in depth to identify the metabolic pathways that were more relevant to the experimental treatment. The screening conditions were enrichment analysis *P* < 0.05 and topological analysis. The influence value was greater than 0.1. As shown in Fig. 7F and G, enrichment analysis of the differential metabolites combined with topological analysis showed that, compared with the cage group, the significantly enhanced metabolic pathways in the ileal content of ground litter broilers mainly included tryptophan metabolism, arginine and proline metabolism, phenylalanine, tyrosine and tryptophan biosynthesis, pyrimidine metabolism, 2-oxocarboxylic acid metabolism, biosynthesis of amino acids, glycerophospholipid metabolism, vitamin B_6_ metabolism, biosynthesis of unsaturated fatty acids, etc.

#### Ileal contents short-chain fatty acids

As shown in Fig. 7H-J, compared with the cage group, the ileal acetic acid and isobutyric acid levels of broilers in the ground litter group on d 28 were higher (*P* < 0.05).

#### Correlation heat map of differential immune parameters, ileal microbes and metabolites

**Fig. 8:**
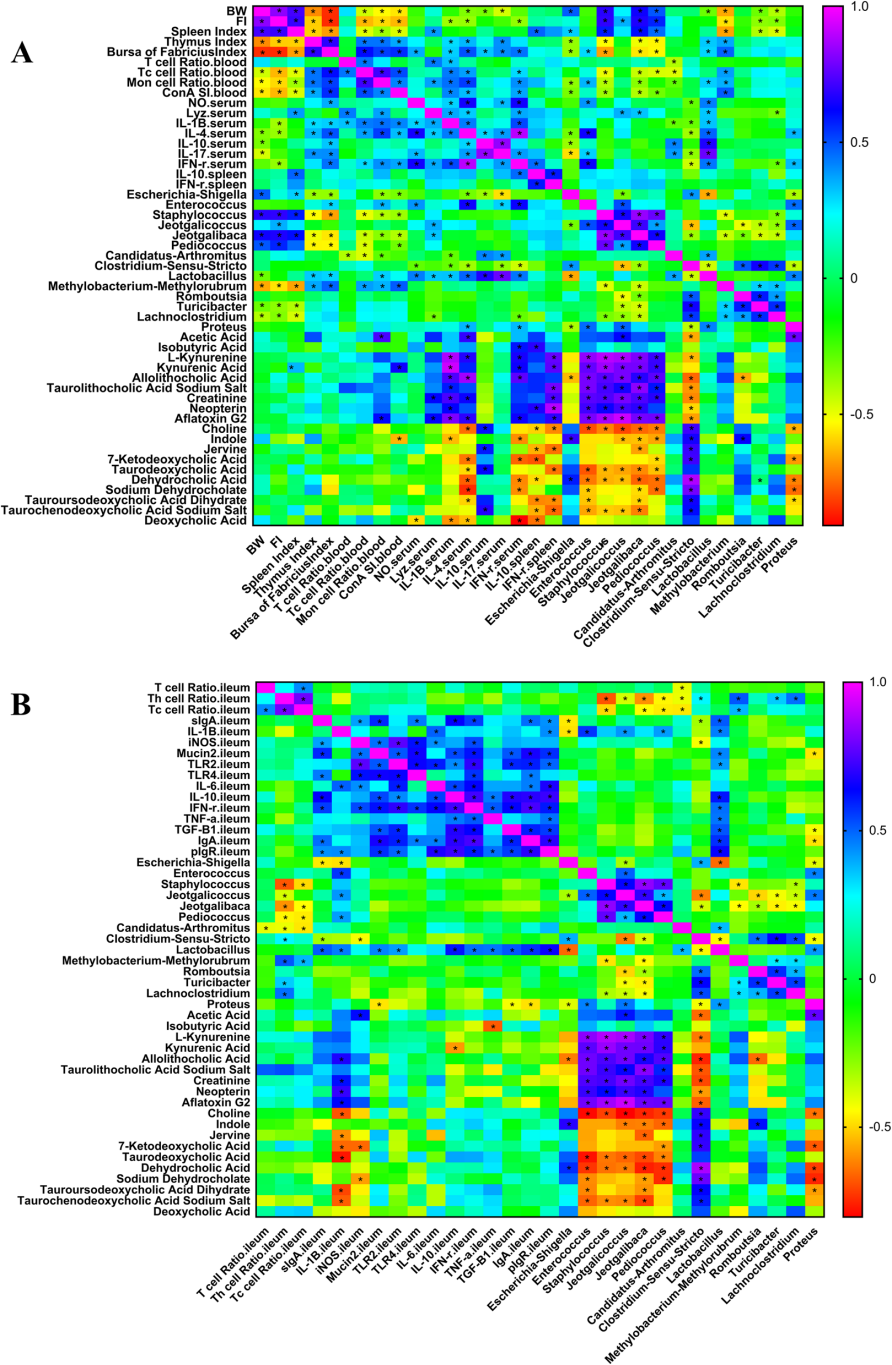
Correlation heat map of ileum differential bacteria and differential immune function of broiler chickens. Spearman’s correlations were calculated for all significantly different peripheral (**A**) and intestinal (**B**) immune parameters and different ileal microbes on genus level. Colors of squares represent *r* values of spearman’s correlation coefficient. ^*^P < 0.05

To explore the relationship between the gut microbiota, metabolites and immune parameters of broilers, based on the abovementioned ileal microbiome and metabolomics data, we then performed a spearman correlation analyze (Fig. 8A and B) to identify the microbes and metabolites related to the stronger immune function of the ground litter broilers. As shown in Fig. 8A, average body weight, feed intake and spleen index were positively correlated with *Escherichia-Shlgella*, *Staphylococcus*, *Jeotgalibaca* and *Pediococcus* and negatively correlated with *Methylobacterium-Methylorubrum*, *Turicibacter* and *Lachnoclostridium*. The thymus index, bursa index, peripheral blood Tc cell ratio, mononuclear macrophage ratio and T lymphocyte transformation rate were positively correlated with *Lactobacillus* and *Methylobacterium-Methylorubrum*, and positively correlated with *Escherichia-Shlgella*, *Staphylococcus*, *Jeotgalibaca*, *Pediococcus* and *Candidatus-Arthromitus* negative correlations. Serum NO, lysozyme activity, and IL-1β, IL-4, IL-10, IL-17 and IFN-γ levels were positively correlated with *Methylobacterium-Methylorubrum* and negatively correlated with *Lactobacillus*. Serum IL-1β, serum IL-4, serum IFN-γ levels, spleen *IL-10* and spleen *IFN-γ* mRNA expression were positively correlated with *L*-kynurenine, kynurenic acid, allolithocholic acid, taurolithocholic acid sodium salt, creatinine, neoopterin and aflatoxin G2 and negatively correlated with choline, indole, jervine, 7-ketodeoxycholic acid and other cholic acids. The spleen immune parameters were positively correlated with kynurenic acid. The proportion of peripheral blood macrophages was positively correlated with acetic acid and aflatoxin G2. The conversion rate of peripheral blood T lymphocytes was positively correlated with kynurenic acid and negatively correlated with indole. Serum NO was negatively correlated with deoxycholic acid. Serum IL-10 was positively correlated with choline, jervine, taurodeoxycholic acid and taurodeoxycholic acid sodium salt.

Figure 8B shows that the ratio of Th and Tc cells in the ileum was positively correlated with *Clostridium_sensu_stricto_1*, *Methylobacterium-Methylorubrum*, *Turicibacter* and *Lachnoclostridium*, and negatively correlated with *Staphylococcus*, *Jeotgalicoccus*, *Jeotgalibaca*, *Pediococcus* and *Candidatus_Arthromitus*. The levels of sIgA in the ileal mucosa and *IL-1β, Mucin2, TLR2, IL-10, IFN-γ, TNF-α, TGF-β1, IgA* and *pIgR* mRNA expression were positively correlated with *Lactobacillus*. Ileal *IL-1β* mRNA expression was positively correlated with *Lactobacillus*, allolithocholic acid, creatinine, neopterin and aflatoxin G2 and negatively correlated with choline, jervine, 7-ketodeoxycholic acid, taurodeoxycholic acid, tauroursodeoxycholic acid dihydrate and taurolithocholic acid sodium salt. The expression of the *iNOS* mRNA in the ileum was positively correlated with acetic acid and negatively correlated with *Clostridium_sensu_stricto_1*, 7-ketodeoxycholic acid and sodium dehydrocholate. Ileal *Mucin2, TGF-β1* and *IgA* mRNA expression was positively correlated with *Lactobacillus* and negatively correlated with *Peoteus*. *IL-10* mRNA expression in the ileum was positively correlated with *Lactobacillus* and negatively correlated with kynurenic acid. Ileal *TNF-α* mRNA expression was positively correlated with metabolites and *Lactobacillus* and negatively correlated with wasobutyric acid. *Enterococcus*, *Staphylococcus*, *Jeotgalicoccus*, *Jeotgalibaca* and *Pediococcus* were positively correlated with *L*-kynurenine, kynurenic acid, allolithocholic acid, taurolithocholic acid sodium salt, creatinine, neopterin and aflatoxin G2 and were positively correlated with dehydrcholine, indolecholate acid, tauroocholic acid and sodiumourcholate. There was a negative correlation between dihydrate and tauroursodeoxycholic acid sodium salt. *Proteus* was positively correlated with acetic acid and negatively correlated with choline, 7-ketodeoxycholic acid, dehydrocholic acid, sodium dehydrocholate and tauroursodeoxycholic acid dihydrate. *Clostridium-sensu-stricto* was positively correlated with choline, indole, jervine, 7-ketodeoxycholic acid, taurodeoxycholic acid, dehydrocholic acid, sodium dehydrocholate, tauroursodeoxycholic acid dihydrate and tauroursodeoxycholic acid sodium salt, and acetic acid, *L*-kynurenine, kynurenic acid, allolitholic acid, taurolithocholic acid sodium salt, creatinine, neopterin and aflatoxin G2 were negatively correlated. *Escherichia-Shlgella* was positively correlated with indole and dehydrocholic acid and negatively correlated with allolithocholic acid.

## Discussion

Our previous research found that, compared with double-layer cages, broiler chickens in the ground litter rearing mode have stronger immune function and richer gut microbiota [45]. We speculate that it may be that more abundant intestinal microbes activate the intestinal immune signaling pathway and intestinal mucosal immune function, and that the proinflammatory cytokines produced enter the peripheral blood through the intestinal barrier. Cytokines stimulate peripheral immune organs, leading to a strong state of systemic immune function. To explore the role of gut microbes in regulating the immune function of the host during this process, in this experiment, we added broad-spectrum antibiotics to drinking water to reduce the number of microbes in the gut. Then, we evaluated the changes in the immune function and status of broilers and looked for sensitive microorganisms and their metabolites that may be related to immune function.

The results of our current study showed that compared with the cage group, broilers raised in ground litter group had higher body weight and feed intake at d 10. This may be due to the fact that broilers raised in ground litter pens were able to get vitamin B_12_ produced by fresh rice hulls, which could increase feed intake and result in a higher body weight gain. This result is supported by Li et al. [46]. They found that on d 7 to 28, body weight gain of Ross 308 broilers appears as FRS (floor litter) > NRS (plastic net) > CRS (cage). Our study also found that compared with the cage group, there was no difference in the growth performance of ground litter group at d 10 to 28. It may be that broilers raised in the ground litter pens were able to contact with feces directly for a long period and with higher foot pad inflammation incidence, which might reduce the weight gain from d 10 to 28 [47]. Differential effects of rearing systems on broiler growth performance may be due to changes in gut microbiota and energy allocation.

In the current study, we found that the addition of broad-spectrum antibiotics to the drinking water significantly increased body weight gain of broilers on 1 to 10. This may be that microbes from feed and environment rapidly colonize and multiply in the gut of chicks, and may compete with the host for some of the nutrients in the gut [48]. After chicks ingested broad-spectrum antibiotics through drinking water, this greatly reduced the number of microbes in the gut, which in turn reduced competition between the microbes and the host for nutrients in the gut lumen [49]. However, this difference of antibiotic treatment on growth performance of broilers disappeared at d 28. This may be due to the negative effects of long-term antibiotic intake on the morphological structure of intestinal villi and crypts of broilers [50], resulting in a decrease in the digestive and absorptive capacity of the intestinal tract, which might reduce the weight gain during d 10 to 28. Some research found that microbes can ferment carbohydrates into short-chain fatty acids, convert diets and endogenous nitrogen-containing compounds into ammonia and microbial protein, and synthesize B vitamins. A mature, healthy and stable gut microbiota also plays an important role in the intestinal health of animals [51]. Others also found that, compared with sterile mice transplanted with normal mouse intestinal contents, sterile mice transplanted with ABX-treated mouse intestinal contents have lower weight, shorter length of ileum. The villi are severely atrophied, and Paneth cells produce lower levels of antimicrobial peptide α defensin 5, indicating that the intestinal microbes after ABX treatment are not conducive to the absorption of nutrients in mice [52]. Their findings are also consistent with ours and can provide a good support for our findings.

Pathogens and inflammatory responses are detrimental to the growth performance of animals, they will disrupt the animal's normal physiological functions such as appetite, digestion and absorption, and reduce the animal's nutrient intake [53]. Although, the maintenance and deployment of the immune system may result in energetic or nutritional costs [54]. Moreover, normal gut microbiota may also compete with the host for some nutrients [48]. However, a normal immune response and a healthy and stable gut microbiota structure are beneficial to the growth performance of animals. This may be because the normal immune function of the host can eliminate or reduce the number of pathogenic bacteria, reduce the inflammatory damage caused by toxins secreted by pathogenic bacteria and excessive inflammation, thereby maintaining the normal nutrient intake and digestion and absorption functions of animals [55]. Furthermore, a stable gut microbiota can repel the colonization of pathogenic bacteria [56]. In addition, gut microbiota decomposes the remaining diet that is difficult for the host to digest by producing enzymes and other means, producing short-chain fatty acids, allolithocholic acid and tryptophan metabolites and other metabolites that are beneficial to host cells [57]. Ultimately, the gut microbiota and immune response contribute to an animal's growth performance.

The immune system is the "army" that protects animal life. It functions as defense, surveillance and self-stability and can respond to disease threats. First, we evaluated the systemic immune function of broilers and found that broilers with ground litters had a higher spleen index; higher proportions of peripheral blood T cells, Tc cells and mononuclear macrophages; stronger proliferation activity of peripheral blood T cells; higher serum NO levels and lysozyme activity; higher serum IL-β, IL-4 and IgG levels; and higher spleen *IL-1β, IL-10* and *IFN-γ* mRNA levels. The results showed that compared with cage broilers, ground litter broilers had stronger systemic immune function and might be in a state of mild systemic inflammation. According to other studies, the spleen index of outdoor hens is higher than that of indoor hens [58]. Compared with the indoor intensive rearing mode, the expression of *TLR7* mRNA in the bursa, lung, duodenum, ileum and cecum of free-range ducks outside is significantly higher [59]. These results are basically consistent with the results of this study. Studies of other types of animals have shown that compared with animals that live in captivity, animals that grow in a more natural environment have stronger peripheral immune functions [60]. The ABX treatment of broilers significantly reduced the index of spleen, thymus and bursa of fabricius, the ratio of peripheral blood T cells and mononuclear macrophages, the phagocytic function of peripheral blood mononuclear cells, serum NO and lysozyme activities, serum IL-4, IL-10, IL-17, IFN-γ levels, serum IgG levels, and spleen *IL-10, IFN-γ, TGF-β1* and *IgA* mRNA levels. This study found that ABX treatment suppressed the systemic immune function of broilers. According to reports, the number of intestinal microbes decreased significantly after ABX treatment of mice. The lack of intestinal microbial stimulation would lead to a decrease in the percentage of Tc cells, Treg cells and DC cells in the spleen. At the same time, the levels of the cytokines IFN-γ and IL-17 produced by Th cells and IL-22 and IL-10 also were reduced accordingly [23], which is consistent with our research results. From this point of view, ABX treatment reduces the stimulation of gut microbiota, which also has a certain negative impact on systemic immune function.

The intestinal mucosa is not only a key site for the digestion and absorption of nutrients, but also a natural barrier to exert immune function and prevent infection by pathogenic bacteria. The intestine is the first target organ to be stimulated by gut microbiota. We speculate that more microbes in the intestines of broilers on the ground litter stimulate the mucosal immune response of broilers through direct stimulation of bacterial components and metabolites, and the cytokines produced enter the blood. Stimulating the spleen of the peripheral immune organs leads to a slight systemic immune response. ABX treatment can significantly reduce the number of intestinal microbes, and the reduction in microbial stimulation may inhibit the state of systemic immune function. Therefore, we also evaluated the mucosal immune function of broilers. The results showed that the proportion of T cells, B cell proliferation activity and *IL-1β* mRNA levels in the ileum of ground litter broilers were higher. The results showed that the ileal mucosal immune cells of ground litter broilers were more active and that the mucosal immune function status was stronger. It has been reported that compared with piglets in an isolator, piglets raised outdoors on the farm have higher pro-inflammatory cytokine IL-2 and lower anti-inflammatory cytokine IL-4 [12]. Studies have reported that the expression of *TLR7* mRNA in the duodenum, ileum and cecum of free-range ducks is significantly higher than that of indoor intensive rearing ducks [59]. Compared with cage broilers, the levels of *IL-1β* and *IFN-γ* mRNA in the ileum of ground litter broilers are higher [7], which is consistent with the results of this study. The results showed that ABX treatment resulted in a lower proportion of T cells, Th cells and Tc cells in the ileum of broilers, lower monocyte phagocytic ability, lower ileal mucosal sIgA levels, lower *iNOS, Mucin2, TLR2, TLR4, IL-6* and *IL-10* mRNA levels, and the mRNA levels of *IFN-γ, TNF-α, TGF-β1, IgA, pIgR* and occludin also decreased. The results showed that treatment with broad-spectrum antibiotics had a significant negative impact on the mucosal immune function and immune cell homeostasis of broilers. It has been reported that the number of lymphoid follicles in the small and large intestines of mice treated with vancomycin or colistin was significantly reduced [61]. After treatment with a broad-spectrum antibiotic consisting of neomycin, vancomycin and metronidazole, the G+ bacteria-specific antimicrobial peptide-regenerating islet-derived protein 3γ in the mouse intestine was significantly reduced [62]. However, vancomycin treatment of mice resulted in a decrease in the number of Treg cells in the colon [63]. In the MLN and PP of mice, the proportion of Treg cells is reduced when the gut microbiota is depleted by treatment with broad-spectrum antibiotics [22]. The number of intestinal microbes decreased significantly after ABX treatment of mice. The lack of intestinal microbial stimulation would lead to a decrease in the proportion of Tc cells, Treg cells and DC cells in the small intestine, colon, mesenteric lymph nodes and spleen, and the cytokines IFN-γ and IL produced by Th cells -17, IL-22 and IL-10 also decreased [23], which is consistent with the results of our study. The above results indicate that the intestinal mucosal immune function status of ground litter broilers is stronger and that the intestinal mucosal immune function status is weaker after ABX treatment. This may be related to the stronger stimulation of the immune system by the gut microbiota of ground litter broilers and the reduction of microbial stimulation in ABX treatment is related.

There is an inseparable relationship between animal immune function and gut microbiota. The interaction between the immune function of broilers and the microbiota may depend on the production of a variety of biologically active small molecular metabolites. The gut microbiota produces small molecule active metabolites (SCFAs, secondary bile acids and indole) through de novo synthesis of lipopolysaccharide (LPS)and peptidoglycan (PGN) [64] and the use of host diets to produce small molecule active metabolites (SCFAs, secondary bile acids and indole) [65] and pro-inflammatory cytokines produced by activated intestinal mucosal immune cells that stimulate target organs and target cells through blood circulation, thereby regulating the homeostasis of the immune system. Therefore, we evaluated the gut microbiota.

In each intestinal segment of broilers, the total number and types of cecal microbes are many and relatively stable. To adjust the inflammatory rearing system and ABX treatment and determine whether the number of intestinal microbes can be adjusted up and down to regulate the stimulation of the immune system, we first detected the total demand in the cecum, the number of aerobic bacteria and total anaerobes. The results showed that the total number of cecal aerobes and total anaerobes in bedding floor chickens was higher. After ABX treatment, the number of cecal total aerobes and total anaerobes was significantly reduced. Further observation revealed that there are two rearing systems under normal conditions. The number of cecal microbes was not much different, but ABX treatment reduced the number of cecal microbes in cages to a higher degree than that of bedding floors. Other studies have shown that broiler chickens have more gut microbes and higher diversity than cages, ground litters and free-range rearing with more environmental microbes [31, 66, 67], which is consistent with our research results. ABX treatment includes neomycin, ampicillin, vancomycin and metronidazole, and the scope of inhibition covers Gram-positive bacteria, Gram-negative bacteria, anaerobic bacteria, protozoa and Trichomonas, which are used to reduce the classic broad-spectrum antibiotic protocol for gut microbiota. Studies using this ABX treatment show that the gut microbiota of mice is significantly changed after ABX treatment, including total aerobes, facultative anaerobes and strict anaerobes in feces. The number of 16S rRNA genes was significantly reduced [23, 68, 69]. We speculate that in the ground litter mode, the litter and the ground contain a large number of microbes. The chickens directly contact the ground and peck at the litter, thereby ingesting a large number of microbes, resulting in an increase in the number of total aerobes and total anaerobes in the cecum. Multiple microbial stimulations enhanced the mucosal immune and systemic immune function status of broiler chickens, and ABX treatment weakened microbial stimulation and downregulated the immune function status.

We used 16S rRNA sequencing to detect the microbiota of ileum. The results showed that the ileum microbial α diversity of ground litter broilers was higher, and the β diversity of the two rearing systems was significantly different. The relative abundances of *Escherichia-Shigella*, *Enterococcus*, *Jeotgalicoccus*, *Pediococcus* and *Jeotgalibaca* were higher in the ileum of ground litter broilers, and *Candidatus_Arthromitus* and *Clostridium_sensu_stricto_1* were lower. According to reports, *Escherichia-Shigella* and *Enterococcus* are opportunistic pathogens that may cause intestinal inflammation and are common in the intestines of patients with inflammatory bowel disease (IBD) [70, 71]. *Jeotgalicoccus* is a Gram-positive coccus that is often found in chicken house litter and air [72]. In my previous research, I found that *Jeotgalicoccus* have a significant positive correlation with immune function of broilers [45]. *Pediococcus* is an excellent probiotic candidate isolated from poultry rectum and feces. It has a wide range of pH values, temperatures and osmotic pressures suitable for growth. It is often selected as a probiotic strain in poultry feed [73, 74]. Studies have reported that adding *Pediococcus* pentosaceus to broiler diets significantly increased the SCFA content in the cecum [75]. *Candidatus_Arthromitus* can induce the innate immune response [76], activate Tc cells in the intraepithelial lymphocyte population [77] and specifically induce Th17 cells [78, 79]. It has been reported that *Clostridium_sensu_stricto_1* is increased in the intestines of infants with IgE-mediated food allergy symptoms and is correlated with IgE levels [80]. Atarashi et al. found that *Clostridium_sensu_stricto_1* promotes the proliferation of Tregs in the colonic mucosa [63]. The results of this study indicate that there are higher types of microorganisms in the ileum of litter-floor broilers, and an increase in *Escherichia-Shigella* and *Enterococcus*, the conditional pathogens that may induce enteritis. In addition, the litter breeding bacteria *Jeotgalicoccus*, which is positively related to immune function, increased, and the *Clostridium_sensu_stricto_1* that promotes the proliferation of anti-inflammatory cells Treg decreased. The ileum microbes of ground litter broilers may cause stronger microbial stimulation to the mucosal immune function and improve the immune function status of broilers, which is consistent with the evaluation results of the immune function status of this study. Other studies have found that, compared with cage broilers, the ileum microbial diversity and relative abundance of actinomycetes are higher in ground litter broilers [7]. Compared with cage ducks, the cecum microbial diversity and opportunistic pathogen *Erysipelato clostridium* of ducks on bedding floors are higher [81]. These studies are consistent with the results of this study. Poultry grows more gut microbes in the rearing system with more environmental microbes, and more microbial stimulation may cause the enhancement of the mucosal immune and systemic immune function of broilers. In addition, this study found that the ileum microbial α diversity of broilers was significantly reduced after ABX treatment, and β diversity was significantly different between groups. ABX treatment reduced *Lactobaclillus* in the ileum of cage broilers and increased *unidentified_Chloroplast*, *unidentified_Mitochondria*, *Enterobacter*, *Romboutsia*, *Bifidobacterium* and *Bacillus*. ABX treatment reduces *Lactobaclillus* in the ileum of the ground litter and increases *Streptococcus*, *Proteus*, *Halomonas*, *Brachybacterium* and *Jeotgalibaca*. According to reports, ABX treatment significantly reduced the number of 16S rRNA genes in mouse feces [23, 68, 69]. *Lactobaclillus* is the main microbe in the ileum of broilers, and its abundance in the ileum of normal broilers is generally above 90% [82]. While it only accounts for approximately 30% of cage chickens, after ABX treatment, it accounts for approximately 60%. We speculate that the decrease in relative abundance indicates a decrease in the total number of 16S rRNA genes in ileal microbes. In addition, bacteria do not contain *Mitochondria* and *Chloroplast*s, so *unidentified_Mitochondria* and *unidentified*_*Chloroplast* may be derived from plant cells that feed in the ileal contents. The operation of extracting DNA from the ileum content of different groups is the same, so the amount of *Mitochondria*l and *Chloroplast* DNA of plant cells in the extracted feed should be the same. The increase in the relative abundance of the two in the ABX treatment group may indicate a reduction in the number of 16S rRNA genes. Therefore, we speculate that ABX treatment reduces the number of 16S rRNA genes of broiler ileum microbes and reduces the number of ileal microbes, thereby reducing the stimulation of ileum microbes on the mucosa and systemic immune function of broilers, which is consistent with the results of the immune aspect in this study.

Changes in the microbial microbiota can lead to changes in the metabolism of microbes and the host. The levels of butyric acid and isobutyric acid in the ileum of ground litter broilers were higher, which may be related to the increase in *Pediococcus* in the ileum. According to reports, SCFAs, especially butyrate, can be used as an energy source for colonic epithelial cells [83]. Studies also have found that symbiotic microbes in the posterior half of the intestine of mice use the SCFA butyrate produced by the fermentation of resistant starch in the intestinal content to help the thymus produce Treg cells, and the produced propionic acid can inhibit histone deacetylation. HDAC enhances the production of new Treg cells in peripheral blood [84], which is consistent with the results of this experiment. This shows that the strong immune function status of ground litter broilers may be related to their metabolite SCFAs, in addition to the direct stimulation of microbes.

In addition to SCFAs, we used the nontarget metabolome to measure the differential metabolites of ileal contents and found that, compared with the cage group, the metabolite composition in the ileum of broilers in the ground litter group changed significantly. Among those that increased were kynurenic acid, *L*-kynurenine, allolithocholic acid, tauroursodeoxycholic acid sodium salt, creatinine, anthranilic acid, xanthurenic acid, neopterin, aflatoxin G2, octopine, agmatine, pyridoxine, citric acid, ulic acidoside, etc., and among the 38 metabolites that decreased were choiline, indole, deoxycholic acid and its salts. Metabolic pathway analysis found that compared with the cage control group (CC), differential metabolites in the ground litter control group (GC) were enriched in the KEGG database with 34 significantly different metabolic pathways, including biosynthesis of unsaturated fatty acids, primary bile acid biosynthesis, tryptophan metabolism, retinol metabolism, arginine biosynthesis, taurine and hypotaurine metabolism, citrate cycle (TCA cycle), vitamin B_6_ metabolism, etc. The correlation analysis results of this study indicate that most peripheral immune functions are positively correlated with *Lactobaclillus*, *Jeotgalibaca*, *Methylobacterium-Methylorubrum*, *Pediococcus*, kynurenic acid, *L*-kynurenine, allolithocholic acid, taurolithocholic acid, creatinine, neopterin and aflatoxin G2, while *Candidatus-Arthromitus*, indole and deoxycholic acid are negatively correlated. Most of the intestinal mucosal immune functions are positively correlated with *Lactobacillus*, allolithocholic acid, creatinine, neopterin and aflatoxin G2, and negatively correlated with *Clostridium_sensu_stricto_1*, 7-ketodeoxycholic acid, taurodeoxycholic acid, sodium dehydrocholate, choline and jervine. The proportion of acetic acid and peripheral blood macrophages was positively correlated with *iNOS* mRNA expression in the ileum. *Jeotgalicoccus*, *Jeotgalibaca* and *Pediococcus* were positively correlated with kynurenic acid, allolithocholic acid, creatinine, neopterin and aflatoxin G2, and negatively correlated with indole, choline, taurodeoxycholic acid and dehydrocholic acid. Previous studies have found that intestinal microbes such as *Clostridium reuteri*, *Lactobaclillus* *reuteri* and *Bacteroides* can metabolize tryptophan to produce indole propionic acid and tryptamine, which can then be transformed into indole, indole lactic acid, indole-3-acetic acid [85–87]. Studies have found that tryptophan metabolites (kynurenine, serotonin and melatonin) and bacterial Trp metabolites (indole, indole acetic acid, skatole and tryptamine) have an effect on the composition of gut microbiota and microbial metabolism. The host immune system, the host-microbiome interface and the host immune system-gut microbiota interaction have a profound impact [88]. Kynurenine, an endogenous metabolite of tryptophan, can endogenously exacerbate colitis by regulating the expression of intestinal NLRP3 inflammasomes [89]. According to reports, indole and its derivative indole acetic acid are products of bacteria metabolizing tryptophan, which can reduce inflammation and maintain intestinal homeostasis [87]. Indole can activate AhR receptors and promote the production of local IL-22, promote the epithelial barrier function of intestinal cells, inhibit the activation of NF-κB and the production of proinflammatory chemokines, and increase the production of anti-inflammatory cytokines, thereby reducing inflammation [88]. The indole derivatives IAA, IPA and ILA can enhance intestinal immune cells and barrier integrity by activating pregnane X receptor (PXR) or AhR receptor [87]. According to reports, bile acids are synthesized in the liver, processed and modified, secreted into the duodenum, and then converted into secondary bile acids under the action of gut microbiota. There are two secondary bile acids: lithocholic acid and deoxycholic acid. They can regulate immune cells, reduce proinflammatory factors, reduce systemic inflammation and alleviate the occurrence of IBD. The study found that murine intestinal symbiotic bacteria (*Bacteroides polymorpha*, *Bacteroides fragilis*) affect the composition of the intestinal bile acid pool and regulate the number of colonic Treg cells. Supplementing SPF mice with inadequate diet and nutrition with a specific combination of primary or secondary bile acids can increase the proportion of colonic Treg cells via the bile acid-VDR axis and reduce the susceptibility to colitis [90]. Studies also have found that centenarians have a unique gut microbiota, which is rich in microbes that can produce unique secondary bile acids, including various isoforms of lithocholic acid. The metabolism of these specific bile acids may be involved in reducing the risk of pathogen infection, which may help maintain intestinal homeostasis [91]. The results showed that the composition of metabolites in the ileum content of ground litter broilers changed significantly. Acetic acid, butyric acid, kynurenine, allolithocholic acid, endogenous tryptophan and microbial metabolites (indole acetic acid, kynuria acid, indole) etc., activate the intestinal mucosal immune function by regulating intestinal cells AhR, NLRP3, GRP41, TLR-NF-κB, etc., leading to mild intestinal inflammation, and this may increase intestinal permeability and cause systemic inflammation.

We speculate that more microbes in the intestines of broilers on the ground litter directly stimulate the mucosal immune response of broilers through bacterial components and metabolites. The produced cytokines and immunomodulatory metabolites enter the blood and stimulate the spleen of the peripheral immune organs and a slight systemic immune response. Broad-spectrum antibiotic treatment can significantly reduce the number of intestinal microbes and reduce the stimulation of the immune system by microbes and metabolites, thereby reducing the immune response of broilers and weakening the immune function status. The poor immune function of cage broilers may be due to the lack of stimulation of environmental microbes and metabolites, and dietary treatments need to regulate their immune function and gut microbiota to enhance their disease resistance.

## Conclusion

In summary, ground litter broilers have stronger immune function, and there were more potential pathogens, litter breeding bacteria, short-chain fatty acids, kynurenine, allolithocholic acid and tryptophan metabolites in the ileum, which were positively correlated with immune function. This may be the reason for its stronger immune function. Broad-spectrum antibiotic treatment leads to a decrease in the proportion of immune cells and cytokine levels in the peripheral and ileal mucosa, and these are related to fewer microbes, such as *Lactobaclillus*.

## Supplementary Information

**Additional file 1: Table S1.** Ingredients and composition (calculated and analyzed nutrients) of the experimental diets1 (%, unless otherwise noted, as-fed basis).

**Additional file 2: Table S2.** Sequences of the oligonucleotide primers used for quantitative real-time PCR.

**Additional file 3: Table S3.** The effect of ABX treatment on the growth performance of broiler chickens in cages and ground litter pens.

**Additional file 4: Table S4.** Up-regulated and down-regulated metabolites in ileum contents from group GC vs. CC (Positive ions.

**Additional file 5: Table S5.** Up-regulated and down-regulated metabolites in ileum contents from group GC vs. CC(Negative ions).

**Additional file 6: Table S6.** Annotation of KEGG pathways of differential metabolites in ileum contents from group GC vs. CC.

**Additional file 7: Fig. S1.** Top ten microbes in the ileum at the genus level of broiler chickens.

**Additional file 8: Fig. S2.** PCA scatter plot for metabolites in ileum contents from group GC vs CC.

## Data Availability

The 16S rRNA gene sequencing data generated and analyzed during the current study are available in the NCBI primary data archive (PDA) with accession number PRJNA 775,677. This data can be found here: https://www.ncbi.nlm.nih.gov/bioproject/775677

## Abbreviations

ABX: Antibiotic
BW: Body weight
BWG: Body weight gain
Con A: Concanavalin A
DTT: 1,4-*DL*-Dithiothreitol
ELISA: Enzyme-linked immuno sorbent assay
FBS: Fetal bovine serum
FCR: Feed conversion ratio
FI: Feed intake
GAPDH: Glyceraldehyde-3-phosphate dehydrogenase
IFN-γ: Interferon-γ
IgA: Immunoglobulin A
IL-1β: Interleukin-1β
iNOS: Inducible nitric oxide synthase
LPS: Lipopolysaccharide
MTT: A 3-(4,5-dimethylthiazol)-2,5-diphenyltetrazolium bromide
NE: Necrotic enteritis
NF-κB: Nuclear factor-κB
NO: Nitrogen oxide
PBMC: Peripheral blood mononuclear cell
pIgR: Polymeric immunoglobulin receptor
PLS-DA: Partial least squares discrimination analysis
RT-PCR: Reverse transcription-rolymerase chain reaction
sIgA: Secretory immunoglobulin A
Tc cell: Cytotoxic T cell
TGF-β: Transforming growth factor-β
Th cell: Helper T cell
TLR2: Toll-like receptor
TNF-α: Tumor necrosis factor α
Treg cell: Regulatory cell
ZO-1: Zonula occludens-1
