# Supplementary material for: Effects of rearing system and antibiotic treatment on immune function, gut microbiota and metabolites of broiler chickens
Source: J Anim Sci Biotechnol. 2022 Dec 16;13:144. doi: 10.1186/s40104-022-00788-y (PMC9756480; doi:10.1186/s40104-022-00788-y)
Supplement: Supplementary file 1 — Additional file 1: Table S1. Ingredients and composition (calculated and analyzed nutrients) of the experimental diets1 (%, unless otherwise noted, as-fed basis). [file 40104_2022_788_MOESM1_ESM.docx]

| **Table S1** Ingredients and composition (calculated and analyzed nutrients) of the experimental diets^1^ (%, unless otherwise noted, as-fed basis) | | |
| --- | --- | --- |
| Item | d 1 to 21 | d 22 to 28 |
| Composition, % | | |
| Corn (7.8% CP) | 51.38 | 60.02 |
| Soybean meal (46% CP) | 40.71 | 25.54 |
| Corn protein flour | 0.00 | 5.66 |
| Soybean oil | 3.75 | 3.32 |
| Wheat flour | 0.00 | 2.00 |
| CaHPO_3_▪2H_2_O | 1.86 | 1.33 |
| Stone powder (37%) | 1.24 | 1.14 |
| Sodium chloride | 0.35 | 0.35 |
| *DL*-Methionine (98%) | 0.20 | 0.070 |
| *L*-Lysine HCL (98%) | 0.00 | 0.19 |
| Vitamin premix^2^ | 0.03 | 0.03 |
| Mineral premix^3^ | 0.20 | 0.20 |
| Choline chloride (50%) | 0.25 | 0.16 |
| Sandoquin (Ethoxyquinoline) | 0.030 | 0.00 |
| Calculated nutrient levels^4^ | | |
| Metabolizable energy, Kcal/kg | 2928.97 | 3100.00 |
| Crude protein | 21.76 | 20.00 |
| Calcium | 1.01 | 0.90 |
| Available phosphorus | 0.44 | 0.35 |
| Lysine | 1.14 | 1.00 |
| Methionine | 0.54 | 0.40 |
| ^1^Diets were in mash form |  |  |
| ^2^Vitamin premix provided per kg of complete diet: vitamin A (retinylacetate), 9500 IU; vitamin D_3_ (cholecalciferol), 2500 IU; vitamin E (*DL*-α-tocopherol acetate), 30 IU; vitamin K_3_(menadione sodium bisulfate), 2.65 mg; vitamin B_12_ (cyanocobalamin), 0.025 mg; biotin, 0.30 mg; folic acid, 1.25 mg; nicotinic acid, 50 mg; *D*-pantothenic acid, 12 mg; pyridoxine hydrochloride, 6.0 mg; riboflavin, 6.5 mg; thiamine mononitrate, 3.0 mg. | | |
| ^3^Mineral premix provided per kg of complete diet: iron, 80 mg; copper, 8 mg; manganese, 100 mg; zinc, 80 mg; iodine, 0.35 mg; selenium, 0.15 mg | | |
| ^4^Calculated value based on the analysis of experimental diets | |  |
