# Supplementary material for: Effects of rearing system and antibiotic treatment on immune function, gut microbiota and metabolites of broiler chickens
Source: J Anim Sci Biotechnol. 2022 Dec 16;13:144. doi: 10.1186/s40104-022-00788-y (PMC9756480; doi:10.1186/s40104-022-00788-y)
Supplement: Supplementary file 2 — Additional file 2: Table S2. Sequences of the oligonucleotide primers used for quantitative real-time PCR. [file 40104_2022_788_MOESM2_ESM.docx]

| **Table S2** Sequences of the oligonucleotide primers used for quantitative real-time PCR^1^ | | |
| --- | --- | --- |
| Gene^2^ | Primer sequence^3^ (5′ to 3′) | GenBank accession No. |
| *GAPDH* | F: AGAACATCATCCCAGCGTCC | NM_204305 |
|  | R: CGGCAGGTCAGGTCAACAAC |  |
| *iNOS* | F: GAACAGCCAGCTCATCCGATA | U34045 |
|  | R: CCCAAGCTCAATGCACAACTT |  |
| *Mucin-2* | F: TTCATGATGCCTGCTCTTGTG | XM_421035 |
|  | R: CCTGAGCCTTGGTACATTCTTGT |  |
| *TLR2* | F: ACCTTCTGCACTCTGCCATT | NM_204278.1 |
|  | R: TGTGAATGAAGCACCGGTAA |  |
| *TLR4* | F: GATGCATCCCCAGTCCGTG | NM_001030693 |
|  | R: CCAGGGTGGTGTTTGGGATT |  |
| *NF-kB* | F: TGGAGAAGGCTATGCAGCTT | NM_205134.1 |
|  | R: CATCCTGGACAGCAGTGAGA |  |
| *IL-1β* | F: TGGGCATCAAGGGCTACA | NM_204524.1 |
|  | R: CGGCCCACGTAGTAAATGAT |  |
| *IL-4* | F: GTGCCCACGCTGTGCTTAC | NM_001007079.1 |
|  | R: AGGAAACCTCTCCCTGGATGTC |  |
| *IL-6* | F: GATCCGGCAGATGGTGATAA | NM_204628.1 |
|  | R: AGGATGAGGTGCATGGTGAT |  |
| *IL-10* | F: CGCTGTCACCGCTTCTTCA | AJ621614 |
|  | R: TCCCGTTCTCATCCATCTTCTC |  |
| *IFN-γ* | F: AAAGCCGCACATCAAACACA | NM_205149.1 |
|  | R: GCCATCAGGAAGGTTGTTTTTC |  |
| *TNF-α* | F: CCCCTACCCTGTCCCACAA | NM_204267 |
|  | R: TGAGTACTGCGGAGGGTTCAT |  |
| *TGF-β1* | F: GCCGACACGCAGTACACCAAG | NM_001318456.1 |
|  | R: GCAGGCACGGACCACCATATTG |  |
| *IgA* | F: ACCACGGCTCTGACTGTACC | S40610.1 |
|  | R: CGATGGTCTCCTTCACATCA |  |
| *pIgR* | F: ATTTGTCACCACCACAGCCA | NM_001044644 |
|  | R: GAGTAGGCGAGGTCAGCATC |  |
| Occludin | F: AGTTCGACACCGACCTGAAG | NM_205128.1 |
|  | R: TCCTGGTATTGAGGGCTGTC |  |
| *ZO-1* | F: ACAGCTCATCACAGCCTCCT | XM_015278981.1 |
|  | R: TGAAGGGCTTACAGGAATGG |  |
| ^1^Primers designed using Primer Express software (Sangon Biotech, Shanghai, China) | | |
| ^2^GADPH = glyceraldehyde-3-phosphate dehydrogenase; iNOS = inducible nitric oxide synthase; TLR2 = toll-like receptor 2; NF-κB = nuclear factor kappa-β; IL-1β = interleukin-1β; IFN-γ = interferon-γ; TNF-α = tumor necrosis factor α; TGF-β1 = transforming growth factor-β1; pIgR = polymeric immunoglobulin receptor; IgA = immune globulin A; ZO-1 = zonula occludens-1  ^3^F = forward; R = reverse | | |
