# Supplementary material for: Effects of rearing system and antibiotic treatment on immune function, gut microbiota and metabolites of broiler chickens
Source: J Anim Sci Biotechnol. 2022 Dec 16;13:144. doi: 10.1186/s40104-022-00788-y (PMC9756480; doi:10.1186/s40104-022-00788-y)
Supplement: Supplementary file 3 — Additional file 3: Table S3. The effect of ABX treatment on the growth performance of broiler chickens in cages and ground litter pens. [file 40104_2022_788_MOESM3_ESM.docx]

| **Table S3** The effect of ABX treatment on the growth performance of broiler chickens in cages and ground litter pens | | | | | | | | | |
| --- | --- | --- | --- | --- | --- | --- | --- | --- | --- |
| Rearing system | ABX^1^ | d 10  BW^2^ | d 0–10  FI^3^ | d 0–10  BWG^4^ | d 0–10  FCR^5^ | d 28  BW | d 0–28 FI | d 0–28  BWG | d 0–28 FCR |
| Cage | - | 136.03 | 184.03 | 94.60 | 1.36 | 927.70 | 1345.44 | 886.54 | 1.52 |
|  | + | 158.42 | 200.29 | 116.47 | 1.27 | 964.38 | 1373.99 | 922.43 | 1.49 |
| Ground | - | 162.11 | 176.23 | 121.07 | 1.09 | 920.80 | 1397.39 | 879.40 | 1.59 |
|  | + | 168.32 | 175.98 | 127.69 | 1.05 | 952.22 | 1444.79 | 911.59 | 1.59 |
| SEM | | 2.074 | 3.593 | 2.090 | 0.024 | 8.314 | 21.666 | 8.341 | 0.023 |
| Main effect | | | | | | | | | |
| Rearing system | Cage | 147.23 | 192.16 | 105.54 | 1.31 | 946.04 | 1359.72 | 904.48 | 1.51 |
|  | Ground | 165.22 | 176.10 | 124.38 | 1.07 | 936.51 | 1421.09 | 895.49 | 1.59 |
| ABX | - | 149.07 | 180.13 | 107.83 | 1.22 | 924.25 | 1371.42 | 882.97 | 1.55 |
|  | + | 163.37 | 188.14 | 122.08 | 1.16 | 958.30 | 1409.39 | 917.01 | 1.54 |
| *P*-value | | | | | | | | | |
| Rearing system | | <0.001 | 0.037 | <0.001 | <0.001 | 0.573 | 0.172 | 0.596 | 0.090 |
| ABX | | 0.003 | 0.278 | 0.003 | 0.181 | 0.054 | 0.391 | 0.055 | 0.779 |
| Rearing system*ABX | | 0.065 | 0.264 | 0.083 | 0.633 | 0.876 | 0.830 | 0.913 | 0.726 |
| ^1^ABX = Antibiotics  ^2^BW = Body weight  ^3^FI = Feed intake  ^4^BWG = Body weight gain  ^5^FCR = Feed conversion rate | | | | | | | | | |
