# Supplementary material for: Effects of rearing system and antibiotic treatment on immune function, gut microbiota and metabolites of broiler chickens
Source: J Anim Sci Biotechnol. 2022 Dec 16;13:144. doi: 10.1186/s40104-022-00788-y (PMC9756480; doi:10.1186/s40104-022-00788-y)
Supplement: Supplementary file 4 — Additional file 4: Table S4. Up-regulated and down-regulated metabolites in ileum contents from group GC vs. CC (Positive ions. [file 40104_2022_788_MOESM4_ESM.docx]

| **Table S4** Up-regulated and down-regulated metabolites in ileum contents from group GC^1^ vs CC^2^（Positive ions） | | | | | |
| --- | --- | --- | --- | --- | --- |
| Items | Names | Fold change | *P*-values^3^ | VIP^4^ | Molecular weight |
| Up-regulated metabolites | | | | | |
| 1 | Stearoyl Ethanolamide | 16.10 | <0.001 | 2.10 | 327.3 |
| 2 | Erucic acid | 2.49 | <0.001 | 1.40 | 338.3 |
| 3 | 2-Methoxyestrone | 5.31 | <0.001 | 1.88 | 300.2 |
| 4 | 4'-Methoxyacetophenone | 3.66 | <0.001 | 1.60 | 150.1 |
| 5 | 1-[4-hydroxy-3-(3-methylbut-2-en-1-yl) phenyl] ethan-1-one | 2.73 | <0.001 | 1.64 | 204.1 |
| 6 | 5'-S-Methyl-5'-thioadenosine | 3.62 | <0.001 | 1.75 | 297.1 |
| 7 | D-Erythrose 4-phosphate | 2.41 | <0.001 | 1.71 | 200.0 |
| 8 | 2-Ketohexanoic acid | 2.31 | <0.001 | 1.68 | 130.1 |
| 9 | 1-(2,4-dihydroxyphenyl)-2-(3,5-dimethoxyphenyl) propan-1-one | 3.96 | <0.001 | 1.80 | 342.1 |
| 10 | GPK | 11.14 | <0.001 | 1.63 | 322.2 |
| 11 | GPH | 89.39 | <0.001 | 1.99 | 347.1 |
| 12 | 1,3-Dimethyluracil | 2.45 | <0.001 | 1.69 | 140.1 |
| 13 | Oleanolic acid | 3.61 | <0.001 | 1.65 | 438.3 |
| 14 | Tyramine | 2.44 | <0.001 | 1.66 | 137.1 |
| 15 | Gelsemine | 10.44 | <0.001 | 1.73 | 322.2 |
| 16 | 2-(4-aminophenoxy) isophthalonitrile | 2.67 | <0.001 | 1.38 | 235.1 |
| 17 | 5-acetyl-2,6-dimethyl-1,2,3,4-tetrahydropyridin-4-one | 2.49 | <0.001 | 1.71 | 167.1 |
| 18 | L-Kynurenine | 8.77 | <0.001 | 1.93 | 208.1 |
| 19 | Lagochilin | 2.40 | <0.001 | 1.41 | 373.3 |
| 20 | Androsterone | 1.65 | <0.001 | 1.28 | 290.2 |
| 21 | N-Acetyl-L-carnosine | 2.63 | <0.001 | 1.76 | 268.1 |
| 22 | D-Ala-D-Ala | 2.02 | <0.001 | 1.51 | 160.1 |
| 23 | 2-(2,6-dimethoxyphenyl)-5,6-dimethoxy-4H-chromen-4-one | 1.96 | <0.001 | 1.67 | 324.1 |
| 24 | Trehalose 6-phosphate | 7.00 | <0.001 | 1.55 | 422.1 |
| 25 | Ergosta-5,7,9(11),22-Tetraen-3-beta-Ol | 3.40 | <0.001 | 1.74 | 394.3 |
| 26 | Adenine | 2.42 | <0.001 | 1.60 | 135.1 |
| 27 | N-(2-hydroxy-2-phenylethyl)-N'-(2-thienyl) urea | 2.18 | <0.001 | 1.62 | 240.1 |
| 28 | 3-(4-hydroxy-3-methoxyphenyl) propanoic acid | 2.68 | <0.001 | 1.70 | 218.1 |
| 29 | O-Desmethylnaproxen | 2.40 | <0.001 | 1.61 | 216.1 |
| 30 | indole-5,6-quinone | 2.05 | <0.001 | 1.28 | 147.0 |
| 31 | 3-(propan-2-yl)-octahydropyrrolo[1,2-a] pyrazine-1,4-dione | 2.32 | <0.001 | 1.53 | 179.1 |
| 32 | Cytidine | 1.99 | <0.001 | 1.56 | 243.1 |
| 33 | 2,4-Dimethylbenzaldehyde | 1.59 | <0.001 | 1.71 | 134.1 |
| 34 | Tiglic acid | 3.65 | <0.001 | 1.71 | 100.1 |
| 35 | LysoPC 12:1 | 2.55 | <0.001 | 1.77 | 433.2 |
| 36 | 12,13-EODE | 4.21 | <0.001 | 1.59 | 296.2 |
| 37 | Dehydroepiandrosterone (DHEA) | 6.85 | <0.001 | 1.89 | 270.2 |
| 38 | 1-benzyl-3-butyl-4-hydroxy-6-phenylpyridin-2(1H)-one | 10.29 | <0.001 | 1.83 | 355.2 |
| 39 | 4-Hydroxyisoleucine | 2.12 | <0.001 | 1.48 | 147.1 |
| 40 | Creatine | 2.83 | <0.001 | 1.52 | 131.1 |
| 41 | N-(1,3-benzodioxol-5-ylmethyl)-6-morpholinonicotinamide | 11.42 | <0.001 | 1.54 | 341.1 |
| 42 | DL-methionine sulfoxide | 1.98 | <0.001 | 1.66 | 182.1 |
| 43 | Asiaticoside | 1.53 | <0.001 | 1.65 | 958.5 |
| 44 | RNK | 4.72 | <0.001 | 1.63 | 416.2 |
| 45 | Azelaic acid | 2.67 | <0.001 | 1.79 | 188.1 |
| 46 | 2-Oxindole | 1.97 | <0.001 | 1.51 | 133.1 |
| 47 | 2-Amino-1,3,4-octadecanetriol | 4.22 | <0.001 | 1.62 | 317.3 |
| 48 | L- (+)-Citrulline | 3.21 | <0.001 | 1.60 | 175.1 |
| 49 | Hydroquinone | 1.62 | <0.001 | 1.48 | 110.0 |
| 50 | 5-S-cysteinyldopa | 1.72 | <0.001 | 1.72 | 316.1 |
| 51 | Ferulic acid | 1.72 | <0.001 | 1.57 | 194.1 |
| 52 | INH | 2.13 | <0.001 | 1.67 | 360.2 |
| 53 | Deoxycorticosterone 21-glucoside | 1.72 | <0.001 | 1.47 | 492.3 |
| 54 | 19(R)-HETE | 1.72 | <0.001 | 1.57 | 320.2 |
| 55 | 4-(4-chlorophenoxy)-3,5-dimethyl-1H-pyrazole | 2.26 | <0.001 | 1.26 | 222.1 |
| 56 | Cuminaldehyde | 1.54 | <0.001 | 1.58 | 148.1 |
| 57 | TKK | 2.08 | <0.001 | 1.63 | 375.3 |
| 58 | Creatinine | 2.42 | <0.001 | 1.50 | 113.1 |
| 59 | Proline-hydroxyproline | 2.17 | <0.001 | 1.29 | 228.1 |
| 60 | 4'-Methoxy-α-pyrrolidinopropiophenone | 2.32 | <0.001 | 1.72 | 233.1 |
| 61 | Allolithocholic acid | 2.73 | <0.001 | 1.61 | 376.3 |
| 62 | DL-Panthenol | 2.13 | <0.001 | 1.51 | 205.1 |
| 63 | o-Cresol | 1.81 | <0.001 | 1.34 | 108.1 |
| 64 | Estrone | 2.12 | <0.001 | 1.59 | 308.1 |
| 65 | 5-Methoxyindole-3-Carbaldehyde | 1.69 | <0.001 | 1.27 | 175.1 |
| 66 | Royal jelly acid | 1.72 | <0.001 | 1.46 | 186.1 |
| 67 | Palmitoyl ethanolamide | 1.96 | <0.001 | 1.33 | 299.3 |
| 68 | 20-Carboxy-Leukotriene B4 | 2.10 | <0.001 | 1.69 | 366.2 |
| 69 | Pyridoxine O-Glucoside | 1.62 | <0.001 | 1.23 | 331.1 |
| 70 | N1, N1-dicyclohexyl-3-(1-naphthyl) acrylamide | 3.04 | <0.001 | 1.58 | 361.2 |
| 71 | 3-Hydroxyanthranilic Acid | 2.05 | <0.001 | 1.66 | 153.0 |
| 72 | Citral | 1.92 | <0.001 | 1.52 | 152.1 |
| 73 | N-acetyl-L-ornithine | 1.85 | <0.001 | 1.45 | 174.1 |
| 74 | N-(5-Aminopentyl) acetamide | 6.89 | <0.001 | 1.26 | 144.1 |
| 75 | 2-(1H-indol-3-yl) acetic acid | 6.26 | <0.001 | 1.40 | 175.1 |
| 76 | Octopine | 1.81 | <0.001 | 1.26 | 246.1 |
| 77 | (R)-3-Hydroxy myristic acid | 4.43 | <0.001 | 1.36 | 261.2 |
| 78 | Testosterone undecanoate | 2.64 | <0.001 | 1.53 | 474.4 |
| 79 | Eicosapentaenoic acid | 1.60 | <0.001 | 1.53 | 302.2 |
| 80 | gamma-Nonanolactone | 2.18 | <0.001 | 1.46 | 156.1 |
| 81 | 5,6-dihydroxyindole | 1.94 | <0.001 | 1.54 | 149.0 |
| 82 | Muramic acid | 1.53 | <0.001 | 1.31 | 251.1 |
| 83 | Tanespimycin | 3.31 | <0.001 | 1.40 | 626.3 |
| 84 | 5-Methylcytosine | 1.63 | <0.001 | 1.20 | 125.1 |
| 85 | 1,7,8-trihydroxy-3-methyl-1,2,3,4,7,12-hexahydrotetraphen-12-one | 2.04 | <0.001 | 1.61 | 310.1 |
| 86 | (12Z)-9,10,11-trihydroxyoctadec-12-enoic acid | 1.63 | <0.001 | 1.51 | 330.2 |
| 87 | 2-(Formylamino)Benzoic Acid | 1.72 | <0.001 | 1.29 | 165.0 |
| 88 | (-)-Caryophyllene oxide | 1.86 | <0.001 | 1.41 | 220.2 |
| 89 | 5-Hydroxyindole | 2.02 | 0.01 | 1.28 | 133.1 |
| 90 | Phe-Pro | 1.98 | 0.01 | 1.27 | 262.1 |
| 91 | 1,2-di(3,4-dimethoxyphenyl) diaz-1-ene | 6.81 | 0.01 | 1.72 | 324.1 |
| 92 | Lysopc 20:0 | 8.94 | 0.01 | 1.35 | 551.4 |
| 93 | IPH | 1.89 | 0.01 | 1.24 | 365.2 |
| 94 | Bisphenol A | 1.61 | 0.01 | 1.58 | 228.1 |
| 95 | Oleamide | 2.12 | 0.01 | 1.44 | 281.3 |
| 96 | 5-allyl-6-methyl-2-(1,4-thiazinan-4-yl) pyrimidin-4-ol | 2.82 | 0.01 | 1.13 | 251.1 |
| 97 | 7,8-Dihydroneopterin | 3.56 | 0.01 | 1.46 | 255.1 |
| 98 | Ouabain | 2.54 | 0.01 | 1.25 | 606.3 |
| 99 | Pyridoxine | 1.51 | 0.01 | 1.53 | 169.1 |
| 100 | Methyl indole-3-acetate | 3.08 | 0.01 | 1.33 | 189.1 |
| 101 | LysoPC 18:0 | 2.11 | 0.01 | 1.39 | 523.4 |
| 102 | YLK | 1.65 | 0.01 | 1.51 | 444.2 |
| 103 | 2-{[(4,5-dimethoxy-2-nitrophenethyl) imino] methyl} phenol | 5.19 | 0.01 | 1.59 | 330.1 |
| 104 | Kynurenic acid O-hexside | 1.65 | 0.01 | 1.23 | 351.1 |
| 105 | N-Acetylhistamine | 1.58 | 0.01 | 1.04 | 153.1 |
| 106 | 4-Hydroxyretinoic Acid | 2.10 | 0.01 | 1.36 | 316.2 |
| 107 | Isoquinoline | 3.12 | 0.01 | 1.35 | 129.1 |
| 108 | cis-7-Hexadecenoic Acid | 1.96 | 0.01 | 1.12 | 254.2 |
| 109 | Anthranilic acid | 1.59 | 0.01 | 1.40 | 137.0 |
| 110 | L-Dopa | 2.01 | 0.01 | 1.47 | 197.1 |
| 111 | Oleoyl ethanolamide | 1.66 | 0.01 | 1.02 | 325.3 |
| 112 | 5-Hydroxyindole-2-carboxylic acid | 4.07 | 0.01 | 1.45 | 177.0 |
| 113 | DL-Norvaline | 1.83 | 0.01 | 1.45 | 117.1 |
| 114 | (5Z)-3-aminonon-5-enoic acid | 102.87 | 0.01 | 1.50 | 171.1 |
| 115 | Levalbuterol | 12.85 | 0.01 | 1.38 | 221.1 |
| 116 | 6-O-Isobutyryl-alpha-D-glucopyranosyl alpha-D-glucopyranoside | 1.55 | 0.01 | 1.29 | 434.1 |
| 117 | Hydroxyamphetamine | 2.76 | 0.01 | 1.48 | 151.1 |
| 118 | 23-Nordeoxycholic acid | 1.84 | 0.01 | 1.33 | 378.3 |
| 119 | Tetrahydrocorticosterone | 2.11 | 0.01 | 1.28 | 350.2 |
| 120 | Tricin 5-O-β-D-glucoside | 2.22 | 0.01 | 1.30 | 492.1 |
| 121 | N~5~-(1,3,5-trimethyl-1H-pyrazol-4-yl)-1H-1,2,4-triazole-3,5-diamine | 14.81 | 0.01 | 1.33 | 207.1 |
| 122 | D-(+)-Camphor | 1.85 | 0.01 | 1.29 | 152.1 |
| 123 | LysoPC 20:2 | 1.67 | 0.01 | 1.01 | 547.4 |
| 124 | N-[2-chloro-6-(trifluoromethoxy) phenyl]-2,2-dimethylpropanamide | 2.83 | 0.01 | 1.20 | 278.0 |
| 125 | 5-Methyl-2'-deoxycytidine | 2.11 | 0.01 | 1.40 | 241.1 |
| 126 | 2-[(3S)-1-Benzyl-3-pyrrolidinyl]-1-methyl-1H-benzimidazole | 10.75 | 0.01 | 1.19 | 291.2 |
| 127 | Anserine | 2.12 | 0.01 | 1.31 | 240.1 |
| 128 | Prostaglandin G2 | 3.74 | 0.01 | 1.32 | 368.2 |
| 129 | D-Glucosamine 6-phosphate | 2.43 | 0.01 | 1.40 | 259.0 |
| 130 | Debromohymenialdisine | 1.61 | 0.01 | 1.18 | 245.1 |
| 131 | Taurolithocholic acid sodium salt | 4.33 | 0.01 | 1.18 | 505.3 |
| 132 | N6-Acetyl-L-lysine | 1.67 | 0.01 | 1.26 | 188.1 |
| 133 | EPK | 2.61 | 0.01 | 1.31 | 186.1 |
| 134 | Bialaphos | 6.95 | 0.01 | 1.19 | 345.1 |
| 135 | Thr-Leu | 1.61 | 0.01 | 1.07 | 232.1 |
| 136 | Orsellinic acid ethyl ester | 2.15 | 0.01 | 1.28 | 196.1 |
| 137 | 5-[(benzoyloxy)methyl]-4,5,6-trihydroxycyclohex-2-en-1-yl benzoate | 1.64 | 0.01 | 1.26 | 401.1 |
| 138 | N6,N6,N6-Trimethyl-L-lysine | 1.66 | 0.01 | 1.21 | 188.2 |
| 139 | Ala-Ile | 1.51 | 0.01 | 1.17 | 202.1 |
| 140 | 2-methyl-2,3,4,5-tetrahydro-1,5-benzoxazepin-4-one | 2.29 | 0.01 | 1.33 | 159.1 |
| 141 | 2,5-Furandicarboxylic acid | 5.64 | 0.01 | 1.38 | 156.0 |
| 142 | Vitamin A | 1.51 | 0.01 | 1.08 | 286.2 |
| 143 | 1-(4-bromophenyl)-2-phenylethan-1-one | 7.94 | 0.02 | 1.34 | 223.1 |
| 144 | (2E)-3-(3,4-dimethoxyphenyl) prop-2-enoic acid | 1.77 | 0.02 | 1.13 | 190.1 |
| 145 | Deoxyinosine | 1.81 | 0.02 | 1.33 | 252.1 |
| 146 | Hexadecanamide | 2.30 | 0.02 | 1.35 | 255.3 |
| 147 | 2-[5-(2-hydroxypropyl) oxolan-2-yl] propanoic acid | 2.09 | 0.02 | 1.49 | 240.1 |
| 148 | 3,4-dihydro-4-oxo-2H-1,3-benzoxazine-2-spiro-4'-(1'-ethylpiperidine) | 6.01 | 0.02 | 1.61 | 422.1 |
| 149 | Neopterin | 1.66 | 0.02 | 1.46 | 253.1 |
| 150 | Sphinganine | 6.59 | 0.02 | 1.40 | 301.3 |
| 151 | N1-cyclooctyl-4-hydroxy-1-piperidinecarbothioamide | 4.01 | 0.02 | 1.10 | 292.2 |
| 152 | Phylloquinone | 2.67 | 0.02 | 1.13 | 450.3 |
| 153 | L-Homocitrulline | 1.50 | 0.02 | 1.28 | 189.1 |
| 154 | Gly-Tyr | 1.66 | 0.02 | 1.24 | 238.1 |
| 155 | N-[(4-hydroxy-3-methoxyphenyl) methyl]-8-methylnonanamide | 1.53 | 0.02 | 1.28 | 329.2 |
| 156 | 4-Ethylbenzaldehyde | 12.48 | 0.02 | 1.42 | 151.1 |
| 157 | RMK | 1.56 | 0.02 | 1.24 | 415.2 |
| 158 | methyl 3,4,5-trihydroxycyclohex-1-ene-1-carboxylate | 1.77 | 0.02 | 1.39 | 170.1 |
| 159 | Tetradecanedioic acid | 4.46 | 0.02 | 1.39 | 258.2 |
| 160 | Coenzyme Q2 | 2.63 | 0.02 | 1.49 | 318.2 |
| 161 | Maleamic acid | 4.98 | 0.02 | 1.30 | 115.0 |
| 162 | D-threo-Isocitric acid | 6.01 | 0.02 | 1.31 | 192.0 |
| 163 | 5-(tert-butyl)-2-methyl-N-(4-nitrophenyl)-3-furamide | 2.60 | 0.02 | 1.09 | 302.1 |
| 164 | (+/-)12(13)-DiHOME | 2.35 | 0.02 | 1.29 | 296.2 |
| 165 | (+/-)5(6)-EET Ethanolamide | 2.72 | 0.02 | 1.51 | 164.1 |
| 166 | trans-Zeatin | 1.84 | 0.02 | 1.29 | 219.1 |
| 167 | Indole-3-acetic acid | 1.97 | 0.02 | 1.21 | 175.1 |
| 168 | DL-Carnitine | 1.59 | 0.02 | 1.16 | 161.1 |
| 169 | PLK | 1.57 | 0.02 | 1.16 | 356.2 |
| 170 | Guanidineacetic acid | 2.57 | 0.02 | 1.43 | 117.1 |
| 171 | (2R)-2-[(2R,5S)-5-[(2S)-2-hydroxybutyl] oxolan-2-yl] propanoic acid | 2.20 | 0.02 | 1.32 | 216.1 |
| 172 | 6,7,8-trimethoxy-2-(2-phenoxy-3-pyridyl)-4H-3,1-benzoxazin-4-one | 9.53 | 0.02 | 1.27 | 444.1 |
| 173 | N-α-L-Acetyl-arginine | 2.15 | 0.03 | 1.27 | 216.1 |
| 174 | N-METHYL (-) EPHEDRINE | 5.67 | 0.03 | 1.25 | 179.1 |
| 175 | 17alpha-Hydroxyprogesterone | 2.25 | 0.03 | 1.18 | 330.2 |
| 176 | Citicoline | 3.36 | 0.03 | 1.16 | 488.1 |
| 177 | 4-Phenylbutyric acid | 1.86 | 0.03 | 1.11 | 164.1 |
| 178 | 9-Oxo-ODE | 1.67 | 0.03 | 1.30 | 294.2 |
| 179 | 5-(2,5-dihydroxyhexyl) oxolan-2-one | 35.74 | 0.03 | 1.34 | 202.1 |
| 180 | O1-[4-(tert-butyl) benzoyl]-2-(tert-butylsulfonyl)ethanehydroximamide | 2.14 | 0.03 | 1.48 | 376.1 |
| 181 | N-[4-(benzyloxy) phenyl]-N'-[2-chloro-6-(4-methoxyphenoxy) benzyl] urea | 14.16 | 0.03 | 1.27 | 526.1 |
| 182 | Xanthurenic Acid | 7.01 | 0.03 | 1.12 | 205.0 |
| 183 | 4-methylphenyl 1-ethyl-3-methyl-1H-pyrazole-5-carbothioate | 2.69 | 0.03 | 1.12 | 260.1 |
| 184 | LNH | 1.54 | 0.03 | 1.09 | 382.2 |
| 185 | Terephthalic Acid | 1.53 | 0.03 | 1.13 | 166.0 |
| 186 | N-Acetyl-D-galactosamine | 1.59 | 0.03 | 1.19 | 221.1 |
| 187 | 2-Arachidonyl Glycerol ether | 2.16 | 0.03 | 1.13 | 342.3 |
| 188 | 3-ethyl-4-hydroxy-1-methyl-1,2-dihydroquinolin-2-one | 2.01 | 0.03 | 1.13 | 203.1 |
| 189 | (11E,15Z)-9,10,13-trihydroxyoctadeca-11,15-dienoic acid | 2.05 | 0.03 | 1.24 | 368.2 |
| 190 | 6-methyl-7-nitro-2,3-dihydro-1,4-benzodioxine | 1.70 | 0.03 | 1.25 | 177.0 |
| 191 | (1E,4E)-1,5-bis(4-methoxyphenyl) penta-1,4-dien-3-one | 1.75 | 0.03 | 1.18 | 316.1 |
| 192 | 1,2-dihydroxyheptadec-16-yn-4-yl acetate | 2.06 | 0.03 | 1.18 | 348.2 |
| 193 | Ergocalciferol | 1.74 | 0.04 | 1.23 | 378.3 |
| 194 | 4-Pregnen-17alpha,20alpha-Diol-3-One | 2.71 | 0.04 | 1.47 | 332.2 |
| 195 | Eicosapentaenoic acid ethyl ester | 2.02 | 0.04 | 1.24 | 347.3 |
| 196 | VPH | 1.62 | 0.04 | 1.20 | 351.2 |
| 197 | Inosine | 1.82 | 0.04 | 1.29 | 268.1 |
| 198 | 15-Deoxy-Δ12,14-prostaglandin A1 | 3.50 | 0.04 | 1.29 | 300.2 |
| 199 | 15-Deoxy-Δ12,14-prostaglandin J2-2-glycerol ester | 3.12 | 0.04 | 1.13 | 408.2 |
| 200 | Pleuromutilin | 3.79 | 0.04 | 1.35 | 400.2 |
| 201 | DL-Citrulline | 1.67 | 0.04 | 1.28 | 175.1 |
| 202 | Riboflavin-5-phosphate | 1.51 | 0.04 | 1.09 | 456.1 |
| 203 | Arachidonoyl amide | 3.50 | 0.04 | 1.33 | 303.3 |
| 204 | Kynurenic acid | 1.81 | 0.05 | 1.14 | 189.0 |
| 205 | Agmatine | 2.78 | 0.05 | 1.12 | 113.1 |
| 206 | Boc-beta-cyano-L-alanine | 1.72 | 0.05 | 1.19 | 214.1 |
| 207 | Υ-L-Glutamyl-L-glutamic acid | 1.96 | 0.05 | 1.09 | 276.1 |
| 208 | N-Cyclohexylformamide | 1.99 | 0.05 | 1.33 | 110.1 |
| 209 | Palmitoleic Acid | 1.64 | 0.05 | 1.11 | 276.2 |
| 210 | VQH | 1.65 | 0.05 | 1.20 | 191.1 |
| 211 | Argininosuccinic acid | 1.53 | 0.05 | 1.08 | 290.1 |
| 212 | Perillartine | 2.89 | 0.05 | 1.08 | 165.1 |
| Down-regulated metabolites | | | | | |
| 1 | Choline | 0.27 | <0.001 | 1.25 | 103.1 |
| 2 | (+)-ar-Turmerone | 0.57 | <0.001 | 1.35 | 234.2 |
| 3 | 7-Ketodeoxycholic acid | 0.17 | <0.001 | 1.12 | 406.3 |
| 4 | Valylproline | 0.50 | 0.01 | 1.49 | 214.1 |
| 5 | Methionine sulfoxide | 0.54 | 0.01 | 1.42 | 165.0 |
| 6 | LPC 16:2 | 0.17 | 0.01 | 1.33 | 491.3 |
| 7 | WLK | 0.41 | 0.01 | 1.52 | 505.3 |
| 8 | L-Phenylalanine | 0.49 | 0.01 | 1.35 | 165.1 |
| 9 | Thymine | 0.40 | 0.01 | 1.72 | 126.0 |
| 10 | Ranolazine | 0.46 | 0.01 | 1.30 | 205.1 |
| 11 | PPK | 0.14 | 0.01 | 1.45 | 322.2 |
| 12 | Taurodeoxycholic Acid | 0.40 | 0.01 | 1.37 | 481.3 |
| 13 | Dehydrocholic acid | 0.38 | 0.01 | 1.75 | 402.2 |
| 14 | (4-benzoylpiperidino) [4-(tert-butyl) phenyl] methanone | 0.46 | 0.02 | 1.30 | 349.2 |
| 15 | KQH | 0.35 | 0.02 | 1.11 | 411.2 |
| 16 | 2'-Deoxyadenosine | 0.47 | 0.02 | 1.52 | 251.1 |
| 17 | Sulfoacetic acid | 0.46 | 0.02 | 1.07 | 140.0 |
| 18 | Desmethylcitalopram | 0.24 | 0.03 | 1.09 | 310.1 |
| 19 | L-Cystathionine | 0.40 | 0.03 | 1.18 | 222.1 |
| 20 | 6-Methylquinoline | 0.60 | 0.03 | 1.19 | 126.0 |
| 21 | Sodium Dehydrocholate | 0.44 | 0.03 | 1.36 | 424.2 |
| 22 | Indole | 0.59 | 0.03 | 1.16 | 117.1 |
| 23 | QQH | 0.10 | 0.03 | 1.24 | 822.4 |
| 24 | 3-Methylcrotonylglycine | 0.61 | 0.04 | 1.21 | 140.0 |
| 25 | FQH | 0.61 | 0.04 | 1.15 | 412.2 |
| 26 | Milbemycin A4 oxime | 0.18 | 0.05 | 1.06 | 555.3 |
| 27 | 3-Amino-4-methylpentanoic acid | 0.61 | 0.05 | 1.34 | 131.1 |
| 28 | Jervine | 0.04 | 0.05 | 1.20 | 425.3 |

^1^GC = ground litter floor control group

^2^CC = cage control group

^3^*P*-values represent the effect of the rearing system

^4^VIP = variable importance in projection
