# Supplementary material for: Effects of rearing system and antibiotic treatment on immune function, gut microbiota and metabolites of broiler chickens
Source: J Anim Sci Biotechnol. 2022 Dec 16;13:144. doi: 10.1186/s40104-022-00788-y (PMC9756480; doi:10.1186/s40104-022-00788-y)
Supplement: Supplementary file 5 — Additional file 5: Table S5. Up-regulated and down-regulated metabolites in ileum contents from group GC vs. CC(Negative ions). [file 40104_2022_788_MOESM5_ESM.docx]

| **Table S5** Up-regulated and down-regulated metabolites in ileum contents from group GC^1^ vs CC^2^（Negative ions） | | | | | |
| --- | --- | --- | --- | --- | --- |
| Items | Name | Fold change | *P*-values^3^ | VIP^4^ | Molecular weight |
| Up-regulated metabolites | | | | | |
| 1 | 5-[4-(2-furylcarbonyl) piperazino]-5-oxo-3-phenylpentanoic acid | 6.68 | <0.001 | 1.91 | 370.2 |
| 2 | Aflatoxin G2 | 4.86 | <0.001 | 1.71 | 330.1 |
| 3 | JWH 250 N-pentanoic acid metabolite | 4.15 | <0.001 | 1.71 | 730.3 |
| 4 | 3,8,9-trihydroxy-10-propyl-3,4,5,8,9,10-hexahydro-2H-oxecin-2-one | 2.37 | <0.001 | 1.76 | 244.1 |
| 5 | Homogentisic Acid | 2.09 | <0.001 | 1.85 | 168 |
| 6 | 15(R)-Lipoxin A4 | 2.51 | <0.001 | 1.77 | 352.2 |
| 7 | Genipin | 4.29 | <0.001 | 1.67 | 208.1 |
| 8 | 2,3-Dinor-TXB2 | 2.23 | <0.001 | 1.46 | 342.2 |
| 9 | L- (-)-Arabitol | 2.06 | <0.001 | 1.76 | 152.1 |
| 10 | (+)-alpha-Lipoic acid | 2.02 | <0.001 | 1.56 | 206 |
| 11 | N-(1,1-Dioxotetrahydro-1H-1λ6-thiophen-3-yl)-4-methoxybenzamide | 3.31 | <0.001 | 1.81 | 269.1 |
| 12 | LPC 14:0 | 1.66 | <0.001 | 1.17 | 527.3 |
| 13 | Quinoline-4-carboxylic acid | 2.33 | <0.001 | 1.39 | 173 |
| 14 | Prostaglandin A3 | 2.55 | <0.001 | 1.51 | 332.2 |
| 15 | 2,4-Dinitrophenol | 7.4 | <0.001 | 1.74 | 368 |
| 16 | Dl-Threitol | 2.39 | <0.001 | 1.5 | 122.1 |
| 17 | 7-Hydroxy-3,4-dihydrocarbostyril | 1.88 | <0.001 | 1.14 | 163.1 |
| 18 | 5-[5-(3-methoxybenzyl)-1,3,4-oxadiazol-2-yl]-2,1,3-benzothiadiazole | 5.18 | <0.001 | 1.94 | 324.1 |
| 19 | 11(E)-Eicosenoic Acid | 8.58 | <0.001 | 1.73 | 310.3 |
| 20 | 7-Methylxanthine | 1.71 | <0.001 | 1.52 | 120 |
| 21 | FAHFA (16:0/18:2) | 6.14 | <0.001 | 1.78 | 534.5 |
| 22 | Citric acid | 3.38 | 0.01 | 1.45 | 192 |
| 23 | N-Acetylneuraminic acid | 1.83 | 0.01 | 1.6 | 309.1 |
| 24 | N-Acetyl-L-glutamic acid | 1.64 | 0.01 | 1.67 | 189.1 |
| 25 | 3-Indoxyl sulphate | 2.37 | 0.01 | 1.38 | 213 |
| 26 | N-[4-(diethylamino)phenyl]-N'-phenylurea | 2.24 | 0.01 | 1.47 | 319.1 |
| 27 | Hydroxyglutaric acid | 1.78 | 0.01 | 1.75 | 148 |
| 28 | N-Acetylvaline | 1.75 | 0.01 | 1.62 | 159.1 |
| 29 | nor-6β-Oxycodol | 1.52 | 0.01 | 1.39 | 303.1 |
| 30 | Methyl-4-hydroxy-3-methoxybenzoate | 1.64 | 0.01 | 1.24 | 182.1 |
| 31 | 5-Methyluridine | 1.87 | 0.01 | 1.6 | 258.1 |
| 32 | Nonanoic acid | 8.2 | 0.01 | 1.6 | 158.1 |
| 33 | trans-10-Heptadecenoic Acid | 2.46 | 0.01 | 1.66 | 268.2 |
| 34 | 2'-Deoxyinosine | 2.86 | 0.01 | 1.63 | 252.1 |
| 35 | Docosanoic Acid | 3.43 | 0.01 | 1.53 | 340.3 |
| 36 | Protoporphyrin IX | 4.38 | 0.02 | 1.39 | 562.3 |
| 37 | Nervonic acid | 3.4 | 0.02 | 1.52 | 366.3 |
| 38 | N1-methyl-5-methoxy-2-({2-[(methylamino)carbonyl] phenyl} thio) benzamide | 3.45 | 0.02 | 1.47 | 331.1 |
| 39 | Lactitol | 1.51 | 0.02 | 1.11 | 344.1 |
| 40 | N-Carbamyl-L-glutamicacid | 1.56 | 0.02 | 1.38 | 190.1 |
| 41 | MGMG (18:2) | 2.91 | 0.02 | 1.38 | 516.3 |
| 42 | 16-Hydroxyhexadecanoic acid | 2.79 | 0.02 | 1.18 | 254.2 |
| 43 | N'1-quinoxalin-2-yl-2-(trifluoromethyl) benzene-1-sulfonohydrazide | 6.24 | 0.02 | 1.71 | 368.1 |
| 44 | Glutaconic acid | 2.07 | 0.02 | 1.63 | 130 |
| 45 | N1-[1-(3-isopropenylphenyl)-1-methylethyl]-3-oxobutanamide | 1.79 | 0.03 | 1.17 | 259.2 |
| 46 | 11(Z),14(Z)-Eicosadienoic Acid | 4 | 0.04 | 1.5 | 308.3 |
| 47 | FAHFA (18:1/20:3) | 5.28 | 0.04 | 1.49 | 586.5 |
| 48 | Elaidic acid | 4.1 | 0.04 | 1.55 | 282.3 |
| 49 | Nonadecanoic acid | 1.82 | 0.04 | 1.3 | 298.3 |
| 50 | 2-Hydroxymyristic acid | 1.7 | 0.05 | 1.16 | 244.2 |
| 51 | Dl-Lanthionine | 1.54 | 0.05 | 1.28 | 208.1 |
| 52 | 4,5-diphenyl-2,3-dihydro-1H-pyrazolo[3,4-c] pyridazin-3-one | 1.64 | 0.05 | 1.04 | 288.1 |
| Down-regulated metabolites | | | | | |
| 1 | 2-(1H-benzimidazol-2-yl)-3-(1,3-benzodioxol-5-yl) acrylonitrile | 0.39 | 0 | 1.58 | 289.1 |
| 2 | Tauroursodeoxycholic acid Dihydrate | 0.25 | 0 | 1.49 | 535.3 |
| 3 | N-Acetyl-Asp-Glu | 0.4 | 0.01 | 1.47 | 304.1 |
| 4 | 1-(4-fluorophenyl)-2-(4-methoxyphenyl)-4-(2-naphthyl) butane-1,4-dione | 0.31 | 0.01 | 1.44 | 412.1 |
| 5 | 5-(tert-butyl)-2-methyl-N-(5-methyl-3-isoxazolyl)-3-furamide | 0.34 | 0.01 | 1.43 | 262.1 |
| 6 | Taurochenodeoxycholic Acid (sodium salt) | 0.41 | 0.02 | 1.25 | 476.3 |
| 7 | Chenodeoxycholic acid-3-beta-D-glucuronide | 0.23 | 0.02 | 1.55 | 568.3 |
| 8 | Glycocholic acid | 0.18 | 0.02 | 1.54 | 465.3 |
| 9 | Deoxycholic acid | 0.27 | 0.03 | 1.32 | 392.3 |
| 10 | Phe-Phe | 0.39 | 0.03 | 1.08 | 312.1 |

^1^GC = ground litter floor control group

^2^CC = cage control group

^3^*P*-values represent the effect of the rearing system

^4^VIP = variable importance in projection
