# Supplementary material for: Effects of rearing system and antibiotic treatment on immune function, gut microbiota and metabolites of broiler chickens
Source: J Anim Sci Biotechnol. 2022 Dec 16;13:144. doi: 10.1186/s40104-022-00788-y (PMC9756480; doi:10.1186/s40104-022-00788-y)
Supplement: Supplementary file 6 — Additional file 6: Table S6. Annotation of KEGG pathways of differential metabolites in ileum contents from group GC vs. CC. [file 40104_2022_788_MOESM6_ESM.docx]

| **Table S6** Annotation of KEGG^1^ pathways of differential metabolites in ileum contents from group GC^2^ vs CC^3^ | | | |
| --- | --- | --- | --- |
| Items | Map Title | *P*-values^4^ | Meta IDs |
| Positive ion mode | | | |
| 1 | Tryptophan metabolism | 0.20 | Kynurenic acid, Indole-3-acetic acid, Anthranilic acid, Indole, Xanthurenic Acid, L-Kynurenine |
| 2 | Arginine and proline metabolism | 0.22 | Octopine, Creatinine, Agmatine, Creatine |
| 3 | Phenylalanine, tyrosine and tryptophan biosynthesis | 0.22 | L-Phenylalanine, Anthranilic acid, Indole, D-Erythrose 4-phosphate |
| 4 | Pyrimidine metabolism | 0.30 | 5-Methylcytosine, Thymine, Cytidine |
| 5 | Ubiquinone and other terpenoid-quinone biosynthesis | 0.40 | Phylloquinone |
| 6 | Oxidative phosphorylation | 0.40 | Riboflavin-5-phosphate |
| 7 | Phosphonate and phosphinate metabolism | 0.40 | Bialaphos |
| 8 | Amino sugar and nucleotide sugar metabolism | 0.40 | D-Glucosamine 6-phosphate |
| 9 | Sphingolipid metabolism | 0.40 | Sphinganine |
| 10 | Glycerophospholipid metabolism | 0.56 | Choline, Citicoline |
| 11 | Vitamin B6 metabolism | 0.56 | Pyridoxine, D-Erythrose 4-phosphate |
| 12 | Pentose phosphate pathway | 1.00 | D-Erythrose 4-phosphate |
| 13 | Fatty acid biosynthesis | 1.00 | Palmitoleic Acid |
| 14 | Steroid biosynthesis | 1.00 | Ergocalciferol |
| 15 | Purine metabolism | 1.00 | Inosine, Deoxyinosine, Adenine, 2'-Deoxyadenosine |
| 16 | Glycine, serine and threonine metabolism | 1.00 | L-Cystathionine, Choline, Creatine |
| 17 | Tyrosine metabolism | 1.00 | L-Dopa, indole-5,6-quinone, Hydroquinone, Tyramine |
| 18 | Taurine and hypotaurine metabolism | 1.00 | Sulfoacetic acid |
| 19 | Retinol metabolism | 1.00 | Vitamin A |
| Negative ion mode | | | |
| 1 | 2-Oxocarboxylic acid metabolism | 0.12 | Citric acid, N-Acetyl-L-glutamic acid |
| 2 | Biosynthesis of amino acids | 0.12 | Citric acid, N-Acetyl-L-glutamic acid |
| 3 | Citrate cycle (TCA cycle) | 0.16 | Citric acid |
| 4 | Biosynthesis of unsaturated fatty acids | 0.24 | Nervonic acid, Docosanoic Aci+A4:I5d |
| 5 | Arginine biosynthesis | 0.30 | N-Acetyl-L-glutamic acid |
| 6 | Caffeine metabolism | 0.30 | 7-Methylxanthine |
| 7 | Alanine, aspartate and glutamate metabolism | 0.30 | Citric acid |
| 8 | Amino sugar and nucleotide sugar metabolism | 0.30 | N-Acetylneuraminic acid |
| 9 | Glyoxylate and dicarboxylate metabolism | 0.30 | Citric acid |
| 10 | Porphyrin and chlorophyll metabolism | 0.30 | Protoporphyrin IX |
| 11 | Ubiquinone and other terpenoid-quinone biosynthesis | 0.42 | Homogentisic Acid |
| 12 | Tyrosine metabolism | 0.42 | Homogentisic Acid |
| 13 | Carbon metabolism | 0.42 | Citric acid |
| 14 | Primary bile acid biosynthesis | 0.51 | Glycocholic acid |
| 15 | Metabolic pathways | 0.70 | N-Acetylneuraminic acid, 7-Methylxanthine, Citric acid, N-Acetyl-L-glutamic acid, Protoporphyrin IX, Glycocholic acid, Homogentisic Acid |

^1^KEGG = kyoto encyclopedia of genes and genomes

^2^GC = ground litter floor control group

^3^CC = cage control group

^4^*P*-values represent the effect of the rearing system
