# Supplementary material for: Effects of rearing system and antibiotic treatment on immune function, gut microbiota and metabolites of broiler chickens
Source: J Anim Sci Biotechnol. 2022 Dec 16;13:144. doi: 10.1186/s40104-022-00788-y (PMC9756480; doi:10.1186/s40104-022-00788-y)
Supplement: Supplementary file 7 — Additional file 7: Fig. S1. Top ten microbes in the ileum at the genus level of broiler chickens. [file 40104_2022_788_MOESM7_ESM.docx]

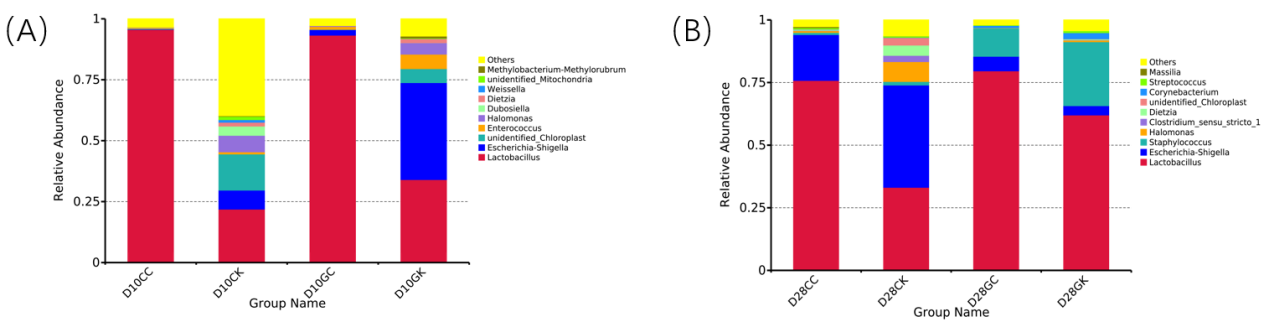
**Fig. S1** Top ten microbes in the ileum at the *genus* level of broiler chickens. CC: cage control group; CK: cage ABX group; GC: ground litter floor control group; GK: ground litter floor ABX group
