# Supplementary material for: Effects of rearing system and antibiotic treatment on immune function, gut microbiota and metabolites of broiler chickens
Source: J Anim Sci Biotechnol. 2022 Dec 16;13:144. doi: 10.1186/s40104-022-00788-y (PMC9756480; doi:10.1186/s40104-022-00788-y)
Supplement: Supplementary file 8 — Additional file 8: Fig. S2. PCA scatter plot for metabolites in ileum contents from group GC vs CC. [file 40104_2022_788_MOESM8_ESM.docx]

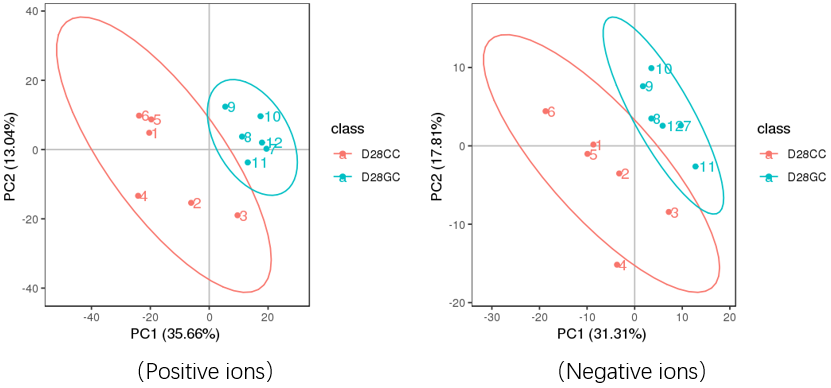
**Fig. S2** PCA scatter plot for metabolites in ileum contents from group GC vs CC. CC: cage control group; GC: ground litter floor control group
